# Development and Application of Reversible and Irreversible Covalent Probes for Human and Mouse Cathepsin‐K Activity Detection, Revealing Nuclear Activity

**DOI:** 10.1002/advs.202401518

**Published:** 2024-07-05

**Authors:** Gourab Dey, Reut Sinai‐Turyansky, Evalyn Yakobovich, Emmanuelle Merquiol, Jure Loboda, Nikhila Sridharan, Yael Houri‐Haddad, David Polak, Simon Yona, Dusan Turk, Ori Wald, Galia Blum

**Affiliations:** ^1^ The Institute for Drug Research The School of Pharmacy The Faculty of Medicine The Hebrew University of Jerusalem Jerusalem 9112001 Israel; ^2^ Department of Biochemistry Molecular and Structural Biology J. Stefan Institute Ljubljana SI‐1000 Slovenia; ^3^ The Institute of Biomedical and Oral Research The Faculty of Dental Medicine The Hebrew University of Jerusalem Jerusalem 9112001 Israel; ^4^ Department of Prosthodontics The Faculty of Dental Medicine The Hebrew University of Jerusalem Jerusalem 9112001 Israel; ^5^ Department of Cardiothoracic Surgery Hadassah Hebrew University Medical Center The Faculty of Medicine The Hebrew University of Jerusalem Jerusalem 9112001 Israel

**Keywords:** activity‐based probes, cathepsin K, covalent probes, imaging protease activity, proteases

## Abstract

Cathepsin‐K (CTSK) is an osteoclast‐secreted cysteine protease that efficiently cleaves extracellular matrices and promotes bone homeostasis and remodeling, making it an excellent therapeutic target. Detection of CTSK activity in complex biological samples using tailored tools such as activity‐based probes (ABPs) will aid tremendously in drug development. Here, potent and selective CTSK probes are designed and created, comparing irreversible and reversible covalent ABPs with improved recognition components and electrophiles. The newly developed CTSK ABPs precisely detect active CTSK in mouse and human cells and tissues, from diseased and healthy states such as inflamed tooth implants, osteoclasts, and lung samples, indicating changes in CTSK's activity in the pathological samples. These probes are used to study how acidic pH stimulates mature CTSK activation, specifically, its transition from pro‐form to mature form. Furthermore, this study reveals for the first time, why intact cells and cell lysate exhibit diverse CTSK activity while having equal levels of mature CTSK enzyme. Interestingly, these tools enabled the discovery of active CTSK in human osteoclast nuclei and in the nucleoli. Altogether, these novel probes are excellent research tools and can be applied in vivo to examine CTSK activity and inhibition in diverse diseases without immunogenicity hazards.

## Graphical abstract

Highly selective reversible‐ and irreversible covalent cathepsin K (CTSK) activity‐based probes are developed and applied to detect CTSK activity in human and mice samples, detecting changes in activity in pathological samples. These non‐toxic probes also enabled studying CTSK activation by pH changes, unique inhibition in cell lysates, and remarkable nuclei localization in human osteoclasts.

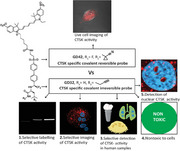

## 1 Introduction

The family of cysteine cathepsin (CTS) proteases, B, C, F, H, K, L, O, S, V, W, and X, have a papain fold and share a preserved catalytic dyad of cysteine and histidine in their active site to degrade their substrates.^[^ ^1^ ^]^ CTS are optimally active at acidic pH and are ubiquitously expressed in the lysosomes, where they maintain homeostasis of various cell types and tissues by their degrading capabilities.^[^ ^2^ ^]^ They are generated as inactive zymogens that are transferred to the lysosome with the assistance of the mannose‐6‐phosphate receptor, where they undergo activation by cleavage.^[^ ^3^ ^]^

Unlike other members of the CTS superfamily, CTSK is most abundantly expressed in giant multinucleated osteoclast cells, actively participates in the degradation of type 1 collagen, and plays important roles in bone remodeling and homeostasis.^[^ ^4^, ^5^, ^6^, ^7^, ^8^ ^]^ During bone remodeling, CTSK is secreted from osteoclasts into the acidified resorption lacunae. After the hydroxyapatite mineral is dissociated in acidic conditions, active CTSK can access and break down the exposed collagen. The overall process results in bone resorption, which is required for normal tissue turnover, existing in concert with osteoblast bone‐building cells. CTSK is highly potent in degrading type‐1 collagen as it can unfold its triple helices and accommodate proline residues in the S2 binding pocket.^[^ ^9^ ^]^ Apart from this, CTSK actively degrades other matrix proteins such as elastin, gelatin, aggrecan, and osteonectin, making it essential for matrix degradation during bone resorption.^[^ ^10^ ^]^

Dysregulation of CTSK may lead to several pathologies, including rheumatoid arthritis, peri‐implantitis, cardiovascular diseases, and several forms of cancer malignancies.^[^ ^11^, ^12^, ^13^, ^14^, ^15^ ^]^ In rheumatoid arthritis, overactivity of CTSK in synovial fibroblasts causes cartilage degradation, eventually leading to joint destruction, and therefore it serves as an important disease biomarker.^[^ ^16^ ^]^ In lung pathology, up‐regulation of CTSK leads to accelerated degradation of elastin during emphysema, while inhibition of the catalytic property of the enzyme results in progressive lung fibrosis.^[^ ^17^ ^]^ In cancer, CTSK is involved in tumor angiogenesis and stromal degradation, facilitating metastasis and tumor growth.^[^ ^12^, ^13^, ^14^, ^15^ ^]^ Specifically, CTSK overexpression in lung cancer cells A549, shows its involvement in the mTOR signaling pathway and in proliferation, migration, and invasion, suggesting its significant prognostic value.^[^ ^18^ ^]^ However, the exact role and mechanism of this enzyme activity in different stages of lung cancer is still unknown due to the lack of appropriate tools that specifically detect CTSK activity. Furthermore, over 3 million people currently suffer from peri‐implantitis‐related problems in the USA alone.^[^ ^19^ ^]^ In most cases, peri‐implantitis bone loss leads to tooth implant loss, due to the lack of a proper diagnosis at the early stages. It is well demonstrated that metalloproteinases and CTSK within the gingival crevicular fluid (GCF) play an important role in matrix destruction and implant loss.^[^ ^20^, ^21^ ^]^ Thus, CTSK activity in GCF fluid may indicate future bone loss. Detection of CTSK activity in various disease samples may shed light on the enzyme's involvement in disease progression and may serve for prognosis.

Several CTSK‐specific inhibitors directed at osteoporosis and osteoarthritis have been developed and tested clinically.^[^ ^22^ ^]^ For example, odanacatib (ODN) was found to be most effective in treating osteoporosis and significantly increased bone mineral density in postmenopausal women.^[^ ^23^ ^]^ Unfortunately, the drug was rejected in phase‐III clinical trials due to the increased risk for cardiovascular morbidity.^[^ ^22^, ^23^ ^]^

Methods for detecting the function and location of CTSK in diseased and healthy tissue may aid in distinguishing whether the adverse effects of ODN and other CTSK inhibitors are caused by off‐target interactions or direct inhibition of CTSK activity. Therefore, we set out to develop probes that selectively identify and demonstrate CTSK activity in situ. For example, detecting CTSK activity within GCF of implant samples can serve as a tool to detect peri‐implantitis before the disease progresses into irreversible stages and implant loss. Reporters of CTSK activity in human tissue may also provide insight into its involvement in disease progression.

Over the years, fluorescent tools to detect CTSK activity have been generated, including substrate probes and activity‐based probes (ABP).^[^ ^24^, ^25^, ^26^, ^27^, ^28^, ^29^ ^]^ The substrate probes play an important role in the assessment of protease activity. Still, they are unable to provide precise information about the localization of the active enzyme, as the cleavage products are detected rather than the location of the protease itself. In addition, due to the high abundance of housekeeping enzymes like CTSL and CTSB along with their high turnover rates for CTSK substrates in most biological settings, it was highly challenging to develop CTSK‐specific probes.^[^ ^30^ ^]^ ABPs, as opposed to substrate probes, covalently link the reporter to the active enzyme enabling detection of the exact location and function level of the enzyme with high spatial resolution. ABPs comprise a small molecule or peptide sequence with selective binding to a target enzyme, an electrophilic warhead that serves as a site for covalent modification by the enzyme active site, and often a fluorescent tag as a reporter. Furthermore, the enzyme‐probe complex enables other biochemical analyses to report on the specific enzymatic activity within the whole proteome. Recently, an acrylamide‐based CTSK ABP was reported in determining CTSK activity in U‐2 OS cells.^[^ ^24^ ^]^ While the probe detects active CTSK, its selectivity was shown to be questionable in complex biological systems. Furthermore, covalent modification generated by ABPs raises potential safety concerns and immunogenicity risks associated with the enzyme's stable modification.^[^ ^31^ ^]^ We, therefore, set out to develop a new class of more selective ABPs that will advance CTSK imaging in live cells and organisms by introducing a target‐specific covalent reversible warhead.

Here covalent CTSK ABPs were generated by linking different warheads, nitrile or acetylene electrophiles, covalent reversible and covalent irreversible (respectively), to an ODN core structure, serving as a recognition element.^[^ ^32^, ^33^, ^34^, ^35^, ^36^ ^]^ Different fluorophores were also introduced, influencing cell‐permeability of the novel CTSK‐specific ABPs. Probes were evaluated as reagents that allow selective detection of CTSK activity in different cell types by comparing their potency, selectivity, and cell permeability. Our newly developed covalent irreversible probe **GD32** was applied in several human and mouse samples with CTSK activity. The probe was used to define the location of CTSK by fluorescent microscopy in mouse bone marrow‐derived osteoclast cells and in human osteoclast cells derived from human peripheral blood mononuclear cells (PBMC) enriched with monocytes. **GD32** was then applied to determine CTSK activity in human lung cancer tissue and normal human lung tissue, where different activity patterns were identified. The probe was also applied to human gingival crevicular fluid (GCF) from inflamed and healthy teeth implants where CTSK detection correlated with the inflammation that leads to implant loss. Furthermore, due to its profound stability in a wide range of pH, **GD32** was applied to study the effect of acidic pH on CTSK activation from its pro form in intact cells. While the covalent irreversible probe **GD32** was found highly potent and selective in all analyses, the covalent reversible probe **GD42** enabled specific CTSK live cell imaging in primary human and mice osteoclasts. These new probes are exciting tools to detect CTSK activity in several sample types and also show promise in future in vivo applications.

## 2 Results and Discussion

**Scheme 1:**
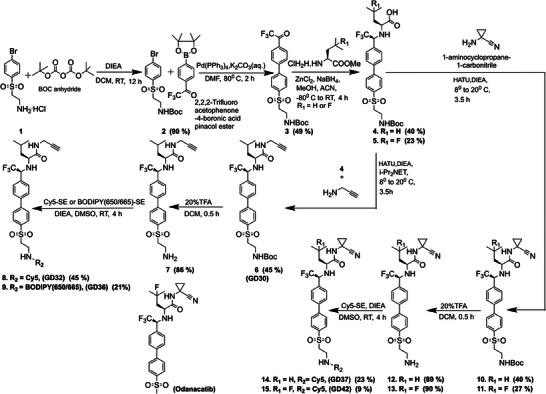
Synthetic route of CTSK ABPs. DIEA, N, N‐Diisopropylethylamine; DCM, dichloromethane; Boc, tert‐butyloxycarbonyl; DMF, dimethylformamide; ACN, acetonitrile HATU, Hexafluorophosphate Azabenzotriazole Tetramethyl Uronium; TFA, trifluoro‐acetic acid; DMSO, dimethyl sulfoxide; BODIPY, Fluorinated Boron‐Dipyrromethene. The structure of Odanacatib (ODN) is presented at the bottom. The overall yield is shown in parenthesis.

To develop novel selective ABPs for CTSK, we modified the structure of ODN to allow both the attachment of a reporter, by adding an amine to the molecule, and introducing various electrophiles as warheads enabling covalent attachment of the probe to CTSK (see the synthetic routes and structures) in **Scheme** **1**, in Supporting Information and Figure S1 (Supporting Information). ODN consists of an electrophilic nitrile group (warhead) that reversibly binds the active site cysteine. The molecule also comprises a cyclopropyl moiety and a binding moiety that includes a methyl‐sulfonyl group see structure in Scheme 1. The inclusion of fluoroleucine in ODN decreases the electron density of the nitrile warhead. As a result, CTSK nucleophilic active‐site cysteine easily attaches to this electrophilic site forming a reversible covalent bond.

Based on ODN, the newly generated ABPs **GD32**, **GD36**, **GD37**, and **GD42** all possess an enlarged methyl‐sulfonyl group that accommodates a fluorophore through an amide bond. In both **GD32** and **GD36**, the nitrile warhead of ODN was replaced with an acetylene (alkyne) warhead. This warhead forms a permanent covalent connection with the cysteine thiol group of CTSK. In addition, the compounds contain a P2 leucine instead of fluoroleucine found in ODN. **GD36** and **GD32** are identical besides having different fluorophores; **GD36** has BODIPY 650/665 while **GD32** has Cy5. **GD37** and **GD42**, similarly to ODN, include a covalent reversible warhead that features a cyclopropyl‐carrying nitrile group. **GD42** and **GD37** differ in structure at the P2 position, **GD37** has a leucine while **GD42** has a fluoroleucine at that position.

The modified core of the probes was synthesized as follows: initially, we protected the amine of the commercial bromobenzyl sulfone ethylamine with a Boc to enable synthesis of the core, and only after that, to attach a fluorescent reporter. In addition, we evaluated the necessity of the fluorinated leucine at the P2 position as electron‐withdrawing fluorine that influences the stability and potency of the electrophilic warhead close by (**GD42** compared to **GD37,** Scheme 1).^[^ ^36^ ^]^ Lastly, in **GD32** and **GD36,** the acetylene warhead was included which permits permanent covalent modification of the irreversible probes. The cyclopropyl derivative of the alkyne was not considered due to its lack of activity toward CTSK, as reported in the literature.^[^ ^34^ ^]^

Synthesis of the probe's recognition elements started by coupling trifluoro acetophenone boronic acid pinacol ester to the Boc‐protected bromobenzyl sulfone ethylamine, by a Suzuki reaction, generating compound **3**. Product **3** was subjected to the formation of the imine intermediates using methyl ester of L‐leucine or 4‐fluoro L leucine followed by diastereoselective reductive amination with NaBH_4_ and ZnCl_2_.^[^ ^37^ ^]^ The resulting mixtures of diastereomeric acids were purified obtaining stereo‐pure forms of the desired acids **4** and **5**. Peptide (amide) coupling reactions of **4** and **5** with the suitable amines generated the various probe cores (**6, 10, 11**). The last step of probe synthesis was Boc removal, followed by the attachment of fluorophores to the amine. We then applied two fluorophores, Cy5 or BODIPY 650/665, both in the near‐infrared region to enable future in vivo applications. **GD42**, **GD37**, and **GD32** contain Cy5, while a BODIPY 650/665 was attached to **GD36** (an analog of **GD32**) to enhance cell permeability and hydrophobicity, as reported for BODIPY dyes.^[^ ^38^, ^39^ ^]^

**Figure 1:**
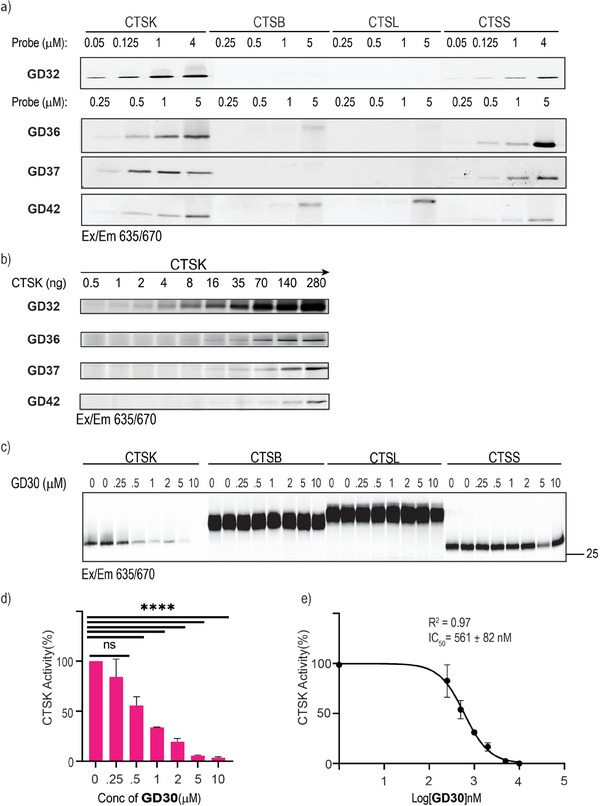
In vitro evaluation of CTSK targeted compounds. a) Labeling of recombinant cathepsins by ABPs. Recombinant cathepsins were treated with increasing concentrations of ABPs for 1 h. Samples were separated by SDS‐PAGE and scanned for fluorescence by a Typhoon scanner at 635/670 nm. All probes show dose‐dependent binding to the CTSK, and have lower affinity to CTSS except **GD36**, b) CTSK [0.5–280 ng] was labeled with the indicated ABPs [5 µm]. Incubations were performed for 1 h at 37 °C, at pH 5.5. Samples were analyzed as described in (a). c) Recombinant cathepsins B, K, L, and S [0.5 µm] were first treated with the indicated concentrations of the inhibitor **GD30** or vehicle for 1 h followed by incubation with a pan cathepsin probe **GB123** [1 µm] for 1 h. Samples were analyzed as described in (a). d) Quantitative analysis of CTSK inhibition by **GD30** presented in **(d)**, data represent mean values ± standard error, n = 3. e) IC_50_ plot of **GD30** inhibition of CTSK activity.

The ABPs were tested for binding to recombinant CTSK compared to the closely related CTSB, CTSL, and CTSS, which are highly abundant and active in most biological samples. Using the in‐gel fluorescent analysis that detects the stable enzyme‐probe complex, active CTSK was labeled with each of the four probes, and significant selectivity was found in three probes **GD32**, **GD37**, and **GD42** (**Figure** **1**a). These three probes only labeled CTSS at higher concentrations, while **GD36** labeled CTSK and CTSS at similar concentrations (Figure 1a). Furthermore, the probes demonstrated high potency by efficiently labeling CTSK even at sub‐stoichiometric concentrations; using the in‐gel fluorescence method **GD32** detected even 0.5 ng of active CTSK. However, the minimum amount of active CTSK the remaining probes detected was much higher, ranging from 8 to 70 ngs, (Figure 1b).

To further evaluate the probes for their selectivity, we applied a potent and selective covalent inhibitor, Compound **6** (**GD30**); the inhibitor was generated as a precursor of **GD32** and **GD36**. **GD30** was evaluated for its ability to block cathepsin activities by preincubating it with each recombinant enzyme before labeling it by a pan cathepsin ABP, **GB123**. **GD30** was found highly selective and potent with an IC_50_ of 561 nm toward recombinant CTSK while not inhibiting the other recombinant cathepsins even at 10 µm (Figure 1c–e).

Since the in‐gel fluorescence method detects the stable complex between the enzyme and the probe, it is expected that the irreversible ABPs (**GD32 and GD36**) show higher efficiency in labeling than the reversible ABPs (**GD37** and **GD42**) not reflecting the actual affinity. We, therefore, continued to examine conditions that enable the detection of reversible ABP binding **GD42**. Indeed, the detection of **GD42** and **GD37** labeling was observed on a gel only when the probes were applied to high amounts of recombinant CTSK. We suspect that only a tiny portion of the probes sustain their binding, as they are fast reversible.^[^ ^34^, ^40^ ^]^ On the other hand, the CTSK‐**GD32** adduct accumulates because of the irreversible nature of **GD32** (Figure S2, Supporting Information). We further tested probe binding in different denaturing conditions. The thioimidate bond formed, as a result of the attack of active protease on the nitrile warhead (covalent reversible), may undergo hydrolysis or elimination at high temperatures (95 °C) or reducing conditions as applied in the sample buffer used in the gel analysis.^[^ ^41^ ^]^ Thus, the stability of the complex between the probes was tested in different denaturing conditions with varying concentrations of beta‐mercapto‐ethanol (BME) within the denaturation loading buffer (Figure S3, Supporting Information).^[^ ^42^ ^]^ While **GD32** was found to be irreversible maintaining the covalent bond after boiling and BME treatments similar to **GB123**, only low levels of **GD42** labeled CTSK remained even after lowering the BME concentrations.

Next, to verify the binding of the reversible **GD42** to CTSK, residual activity was detected by a pan cathepsin ABP **HG122**,**^[^** ^43^ ^]^ labeled with a different fluorophore (BODIPY TMR‐X), that was added to CTSK after preincubation with **GD32** or **GD42**. The cathepsin residual activity was detected by the BODIPY TMR‐X (Cy3 scanning) fluorescent gel scan. The decrease in **HG122** fluorescence intensity was more profound in **GD32** than in **GD42**, reflecting the reversible binding mode of **GD42;** additionally, the direct labeling of the irreversible **GD32** probe is detected in the Cy5 channel (Figure S4, Supporting Information).

We then determined the kinetic inhibition parameters of one presumable irreversible covalent probe **GD32** and one reversible covalent probe **GD37** toward the four closely related cathepsins, CTSB, CTSK, CTSL, and CTSS. Both probes showed significant binding to CTSK while maintaining extremely high selectivity over the other cathepsins. Even for the closely related CTSS, **GD32** and **GD37** were 115 and 150 times less potent, respectively, compared to CTSK, similar to the selectivity of ODN (Table 1).

**Table 1a:** Cathepsin inhibition by irreversible and reversible probes. Cathepsin concentrations in the assay were 0.4 nM (CTSK) or 5 nm (CTSS, CTSB, and CTSL). Fluorescent substrates Z‐RR‐AMC (CTSL) or Z‐FR‐AMC (CTSS, CTSB, and CTSK) were used and detected at excitation 370 nm and emission 460 nm. More details are available in the Methods section. **1b**. Mass spectrometry data showing recombinant CTSK binds **GD32** covalently. Recombinant CTSK was subjected to matrix‐assisted laser desorption/ionization (MALDI) mass spectrometry to identify its exact mass. CTSK was labeled with **GD32** for 5 h and then subjected to MALDI. The half mass of the adduct CTSK‐**GD32** was detected (full mass 12 366).

a. Compound	GD32	GD37	ODN
Cathepsin			
**CTSK**	Irreversible k_inact_/K_i_: 26 ± 14 × 10^4^ M^−1^sec^−1^ k_inact_: 0.0007 ± 0.0001 sec^−1^ K_i_: 3.6 ± 2.6 nm	Reversible [Tight binding] IC_50_ = 0.40 ± 0.2 nm	Reversible [Tight binding] IC_50_ = 0.94 ± 0.86 nm
**CTSS**	Irreversible k_inact_/K_i_: 2220 ± 300 M^−1^sec^−1^ k_inact_: 0.00220 ± 0.00009 sec^−1^ K_i_: 980 ± 170 nm	Reversible K_i_ = 60 ± 28 nm	Reversible IC_50_ = 140 ± 97 nm
**CTSL**	Reversible K_i_ = 20 ± 2.6 µm	Reversible K_i_ = 1.2 ± 0.1 µm	–
**CTSB**	Reversible K_i_ = 9.4 ± 0.8 µm	Reversible K_i_ = 5.9 ± 1.5 µm	–
**b**.	**Exact Mass found [M/Z]**	**Explanation**	
**CTSK**	23598.44	The full mass of CTSK	
**CTSK‐GD32**	12366.19	Half mass of CTSK bound to **GD32** (23598.44+1134.4)/2 = 12366.42	

To verify the covalent nature of **GD32** to CTSK, the enzyme was subjected to matrix‐assisted laser desorption/ionization (MALDI) before and after labeling with the probe. The half mass of the adduct was identified, as **GD32** carries a positive charge within the Cy5 moiety (Table 1). This data supports the efficient labeling of CTSK by **GD32**.

**Figure 2:**
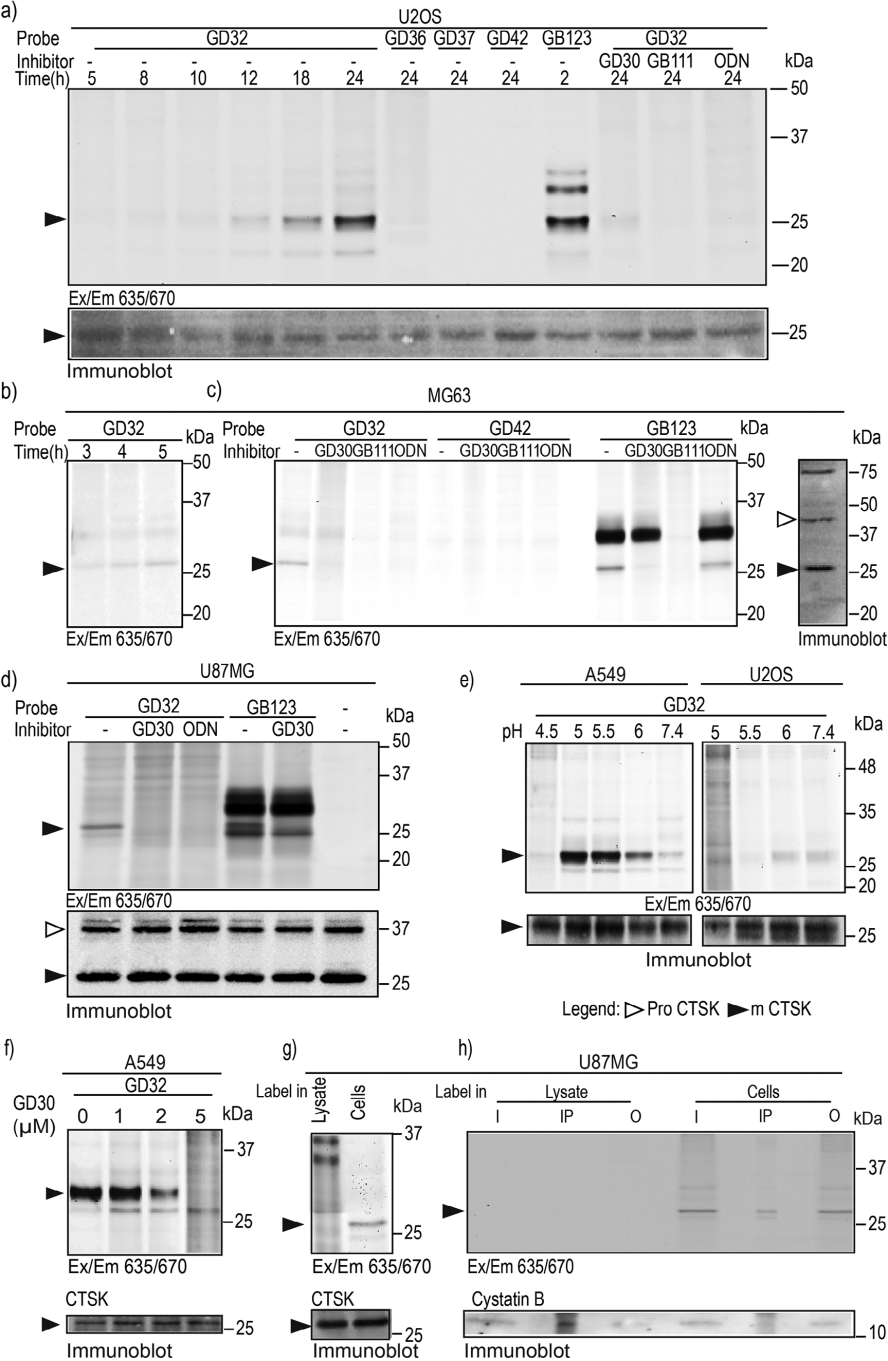
Labeling of endogenous CTSK in intact cells. a) U2‐OS cells were treated with DMSO or 5 µm of the indicated inhibitors in culture medium, for 2 h, then with indicated ABPs [5 µm] in a culture medium for indicated times, followed by cell lysis, SDS‐PAGE separation, and fluorescent scanning of the gel at 635/670 nm. An immunoblot of CTSK of the same gel is presented below. b) Intact MG63 cells were treated with **GD32** 5 µm in the culture media for indicated times, cell lysates were analyzed as in a. CTSK immunoblot is presented, pro and mature CTSK are indicated by arrows. c) MG63 cells and d) U87MG cells were treated with DMSO, or 5 µm of indicated inhibitors in culture medium for 2 h, followed by incubation with 5 µm of indicated probes for 5 h (**GD32** and **GD42**) and 2 h (**GB123**), cell lysates were analyzed by fluorescent SDS PAGE as in a. High selectivity of **GD32** toward CTSK is seen as it does not label other active cathepsins in the cells that are labeled by **GB123**. In (c) a CTSK immunoblot of a representative MG63 lane on the right. e) Labeling of active mature CTSK by **GD32** in A549 and U2‐0S cells in externally adjusted different pH. Cells were incubated with 5 µm **GD32** for 24 h in growth media at indicated pHs, prior to the cell lysis, cell lysates were analysed as in a. CTSK immunoblot below. f) A549 cells were incubated with increasing concentrations of **GD30** for 2 h at pH = 5, followed by the incubation with 5 µm **GD32** for 24 h prior to the cell lysis, cell lysates were analyzed as in a. g) Intact U‐87 MG cells or U‐87 MG cell lysates were treated with 5 µm **GD32** for 2 h, the intact cells were then lysed and all lysates were analyzed as in (a). h) Intact or lysates of U‐87MG were treated with **GD32** for 2 h then immunoprecipitation was done with an anti‐CTSK antibody. Input (I), immunoprecipitation (IP), and output (O) were separated by a 14% SDS‐PAGE and scanned for fluorescence. The gel was then blotted and reacted with an anti‐cystatin B antibody below. **ODN (Odanacatib), GB111 (GB111‐NH_2_), mCTSK (mature CTSK)**.

To test probe selectivity and the ability to label endogenous CTSK activity in intact cells, three cell lines were selected: human osteosarcoma MG‐63 and U‐2 OS, and human glioblastoma U‐87 MG; all cell lines express CTSK along with other lysosomal cathepsins.^[^ ^44^ ^]^ First, the ABPs were incubated with intact U‐2 OS cells for different durations of time, and the probe selectivity toward CTSK was examined, with a pan cathepsin ABP, **GB123**, used as a control that labels CTS B, K, L, and S in cells. After cell lysis, the probe‐labeled enzymes were resolved and detected by a fluorescent scan of the SDS‐PAGE. **GD32** retained its CTSK specificity up to 24 h for U‐2 OS cells, the preincubation of the cells with the pan cathepsin inhibitor, **GB111‐NH_2_**, or with the CTSK specific inhibitors, **GD30** or ODN, resulted in the depletion of the CTSK‐specific band, (**Figure** **2**a). In similar experiments, we demonstrated the probes' specificity and ability to bind CTSK. We also found that various cells required varied incubation durations. Specifically, intact MG‐63 cells required a 5 h incubation (Figure 2b,c), while U‐87 MG cells required a 2 h incubation (Figure 2d). In addition, the labeling of CTSK remained significantly specific even in cells that had low levels of mature CTSK expression but high levels of CTSB and CTSL (such as U‐87 MG, MG‐63 cells) (Figure 2a–d).

The binding of the BODIPY based probe **GD36**, reversible covalent probes **GD42** and **GD37** to active CTSK could not be detected in intact cells using the fluorescent SDS PAGE method, (Figure 2a; Figure S5, Supporting Information). Nevertheless, the fluorescence band near 25 kDa corresponding to active CTSK was observed in both MG‐63, U‐2 OS, and U‐87 MG cells using the covalent irreversible probe **GD32**. Thus, tuning proper probe concentration and incubation time may enable selective imaging of the endogenous CTSK in intact cells even with low amounts of active enzyme. We further corroborated the band identity by western blot with an anti‐CTSK antibody, where the mature active CTSK is detected at the same location on the gel (Figure 2).

The mature version of CTSK actively breaks down type‐II collagen in the acidic pH environment of osteoclasts. This supports the observation that autoactivation of CTSK occurs at acidic pH in the recombinant phase.^[^ ^45^ ^]^

Additionally, it was shown that acidic extracellular pH is a major feature of tumor tissue.^[^ ^46^ ^]^ Consequently, it would be of scientific interest to further explore the impact of acidic pH on the activation of CTSK, specifically the transition from its pro‐form to its mature form, within intact cells. Therefore, the human lung cancer cell line A549, as well as the human osteosarcoma cell line U‐2 OS were used. Cells were cultured in media adjusted with different pHs externally for 24 h acknowledging the uncertainty regarding its correlation with the intercellular pH, and then incubated with **GD32**. **GD32** successfully detected CTSK in a wide range of pH. However, the activity was pH dependent, revealing a different pH optimum for CTSK activation in different cell lines, (Figure 2e). While optimal CTSK activation in A549 cells is at pH 5, in U‐2 OS it is approximately pH 6. Furthermore, the preincubation of A549 cells with increasing concentration of CTSK inhibitor **GD30** at pH 5 blocked the activity of the enzyme (Figure 2f).

In order to examine the utility of CTSK‐specific ABP **GD32**, the U87‐MG cell line was chosen because of its considerable expression of mature CTSK, as shown in^[^ ^44c^ ^]^ (Figure 2d). We show that intact U87‐MG cells have modest CTSK activity, however after lysing the cells, only negligible activity was detected. While both the intact cells and the cell lysate have similar levels of mature CTSK as expected, the difference in CTSK activity between samples is attributed to the binding of the endogenous inhibitor Cystatin B. By co‐immunoprecipitation experiments we show that CTSK binds increased amounts of its inhibitor Cystatin B, as a result of the lysing process (Figure 2g,h). Based on these results, we concluded that CTSK activity is inhibited in lysates but not in intact cells.

**Figure 3:**
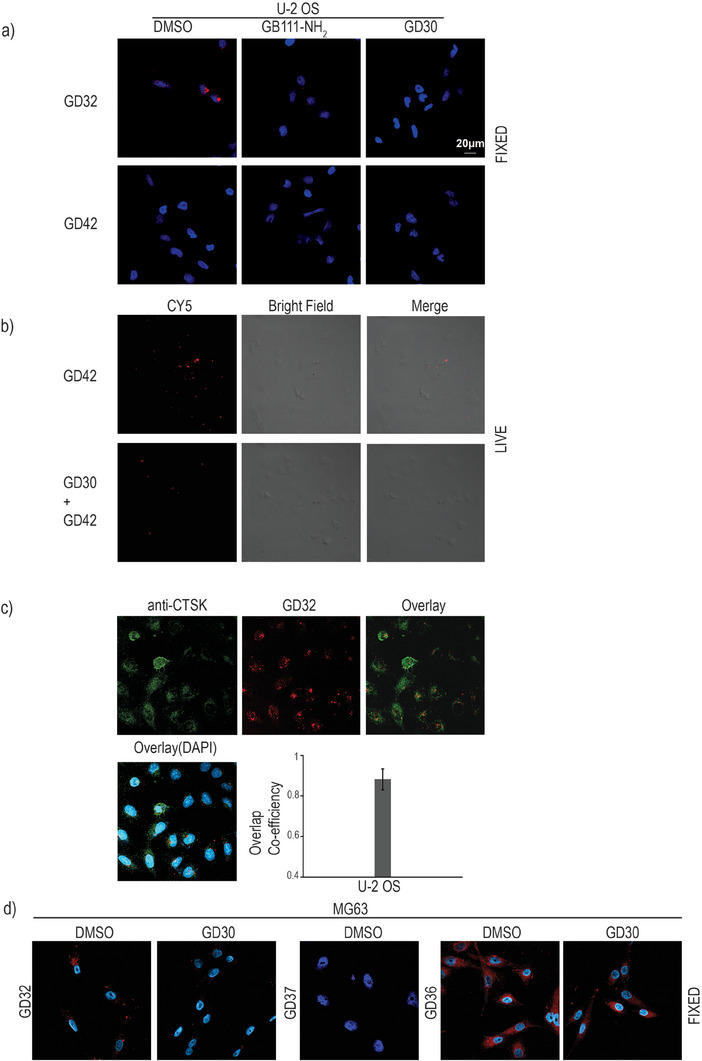
CTSK activity detected in intact cells. a) Cultures of U2‐OS cells were grown in 8‐well chambers. Cells were either pretreated with the pan‐cathepsin inhibitor **GB111‐NH_2_** [3 µm] or CTSK‐specific inhibitor **GD30** [3 µm], or with DMSO vehicle [0.1%] for 2 h and then labeled by the addition of 1.25 µm indicated probe to the culture medium for 6 h, after fixation cells were stained with DAPI (nucleus) and imaged by confocal microscopy. b) Live imaging of U2‐OS cells, cells were either pretreated with inhibitor **GD30** [3 µm] or with DMSO vehicle [0.1%] for 2 h and then labeled with **GD42** (1.25 µm) for 6 h, cells were washed quickly and immediately imaged by confocal microscopy acquired with an Olympus FV10i inverted microscope, representative images are shown. c) The localization of CTSK in U2‐0S cells was determined using ABP **GD32** and an anti‐cathepsin K antibody. The ABP (red signal) and CTSK antibodies (green signal) exhibited a significant overlap, indicating the selectivity of the ABP. d) CTSK activity localization studies in MG63 cells. Cultures of MG63 cells were grown in 8‐well chambers. Cells were either pretreated with the CTSK inhibitor **GD30** [3 µm] or with DMSO vehicle [0.1%] for 2 h and then labeled by the addition of 1.25 µm of the indicated probes to the culture medium for 5 h, after fixation cells were stained with DAPI (nucleus) and imaged by confocal microscopy. Red, probe fluorescence, blue, DAPI.

The probes were then evaluated for their ability to detect active CTSK by fluorescent microscopy. ABPs were incubated with intact U‐2 OS cells, after preincubation with vehicle, a pan cathepsin inhibitor (**GB111‐NH_2_**),^[^ ^47^ ^]^ or our selective CTSK inhibitor **GD30**. Cells were then fixed and imaged by confocal fluorescent microscopy, (**Figure** **3**a). The irreversible **GD32** detected active CTSK and was inhibited by both inhibitors, indicating probe specificity. While the fixation process did not influence the binding of the irreversible probes, it led to the elimination of the reversible probe labeling **GD42**, which resulted in no signal. However, when live cell imaging was performed without fixation, active CTSK was detected by **GD42** although barely, reflecting the probe affinity together with the low level of active CTSK in this cell line (Figure 3b). In order to determine the specificity of **GD32**’s fluorescence signaling, U2‐OS cells were treated with the probe. Then the cells were fixed and labeled with a CTSK antibody labeled with Cy3. The signal from the CTSK labeled by probe in the cells (Cy5) colocalized with the CTSK antibody (Cy3) with (overlap coefficiency of 0.892) (Figure 3c). Additionally, the increased extent of the antibody signal may be attributed to its interaction with the pro‐CTSK, or CTSK that is bound to an endogenous inhibitor, which should not be detected by **GD32** since they are not active.

Labeling of **MG63** cells showed similar results, where the irreversible probe (**GD32**) stayed covalently linked, and the reversible probe, **GD37**, was eliminated, (Figure 3d). While **GD36** a BODIPY tagged probe showed high cell accumulation, it was not specific to cathepsin labeling, as it was not eliminated by inhibitor pretreatment, most likely due to the known non‐specific lipid binding of BODIPYs,^[^ ^48^ ^]^ (Figure 3d). The pan cathepsin ABP **GB123** showed intense labeling of the cathepsins in these cells, though the signaling intensity was completely blocked by the pan cathepsin inhibitor **GB111‐NH_2_** (though not the selective inhibitor) (Figure S6, Supporting Information). These results demonstrate the ability of **GD32**, and **GD42** to image CTSK activity in intact cells.

To further corroborate the selectivity of **GD32,** we tested the labeling of Jurkat cells, which lack mature CTSK. Intact cells were incubated with the probe for different durations up to 24 h before they were lysed and analyzed by fluorescent SDS‐PAGE. As expected, no fluorescent bands were detected by **GD32**, although other active cathepsins were present‐ as seen by the **GB123** labeling (Figure S7, Supporting Information). While pro‐CTSK was detected‐ these cells lack mature CTSK as seen by the immunoblot of cell lysates (Figure S7, Supporting Information bottom). These findings led us to select **GD32** as a lead compound for further investigation of CTSK's activity and location in different biological and medical samples.

**Figure 4:**
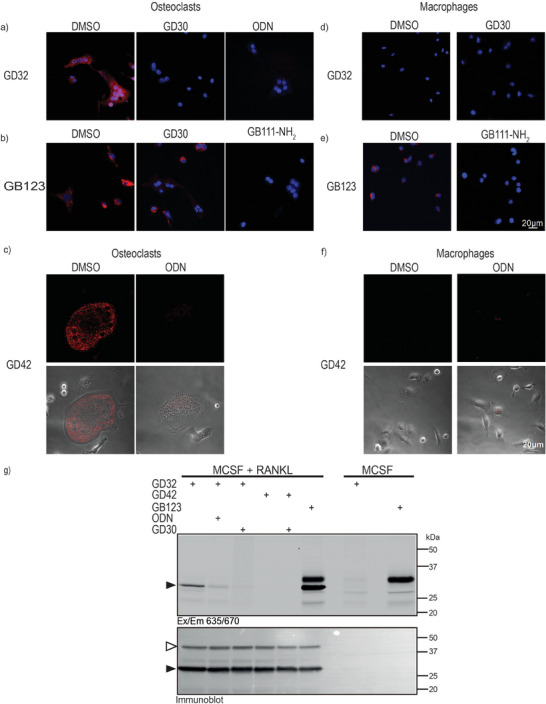
Direct labeling of endogenous CTSK in intact mouse osteoclasts. Mice monocyte cells collected from bone marrow were treated with MCSF and RANKL for nine consecutive days generating osteoclasts (Figure a, b, and c) or with MCSF alone ‐generating macrophages (Figure d, e, and f). Cells were treated with vehicle (DMSO) or inhibitors **GD30,** ODN or **GB111NH_2_ ODN** or **GD30** [3 µm] in a culture medium for 2 h, followed by incubation with **GD32** or **GD42** for 6 h or **GB123** for 2 h. The cells treated with the irreversible probes **GD32** and **GB123**, were fixed, stained with DAPI, and imaged using confocal fluorescence microscopy. Cells treated with **GD42** underwent live cell microscopy. g) CTSK inhibitors and probes (5 µm) were applied to either osteoclasts or macrophages as described in (f). Cell lysates underwent SDS‐PAGE separation, and fluorescent scanning of the gel after which the gel was transferred and western blotted with an anti‐CTSK antibody. The position of mature CTSK is indicated. Red fluorescence marks cathepsin activity (ABPs) and blue fluorescence marks nucleus (DAPI).

Human CTSK is different in structure from mouse CTSK due to three different amino acids.^[^ ^49^ ^]^ The ABPs presented here were designed based on odanacatib, which is human‐specific. We, therefore, tested the cross‐reactivity of **GD32** to both mouse and human CTSK in osteoclasts, knowing that detection of mouse CTSK may aid in pre‐clinical models for drug development. Primary multinucleated osteoclasts were generated from bone marrow cells that were extracted from mice tibia and fibula and then supplemented with RANKL and MCSF to yield osteoclast differentiation as described.^[^ ^50^ ^]^ Osteoclasts were then incubated for 6 h with the irreversible or reversible probes before confocal microscopy. Probe labeling shows punctate CTSK staining resembling lysosome distribution, supporting CTSK's expression pattern as reported in the literature,^[^ ^51^ ^]^ (**Figure** **4**). This finding may also be a result of multinucleated giant cells that utilize the cell surface to execute their bone‐resorbing function by CTSK. Moreover, in the presence of CTSK‐specific inhibitor **GD30,** or ODN, the signaling intensity was abolished in fixed cells, indicating **GD32** specificity toward active CTSK in the osteoclastic milieu (Figure 4a).

Labeling primary mice osteoclasts with the pan cathepsin probe **GB123** in comparison to **GD32**, yielded a more intense signal from binding to additional cathepsins that are active in these cells (Figure 4b). As controls, osteoclasts were preincubated with pan cathepsin inhibitor **GB111‐NH_2_**, or **GD30**, prior to **GB123** labeling, while the pan cathepsin inhibitor eliminated the fluorescent signal, **GD30** only mildly reduced the **GB123** signal. Moreover, the covalent reversible ABP, **GD42,** also selectively labeled active CTSK but had to be detected by live cell imaging without fixation, and the signal was strongly reduced after ODN pre‐treatment (Figure 4c). Furthermore, to confirm the ABP's selectivity toward CTSK, primary macrophages underwent similar analyses with both probes (Figure 4d,f). Macrophages were generated by treatment with MCSF as described in.^[^ ^52^ ^]^ **GD32** does not bind to any macrophage protease, unlike **GB123**, which binds to other cathepsins in the cells, (Figure 4b,e). Analyzed by live cell imaging, **GD42** did not produce any signaling in macrophages due to the absence of active CTSK, unlike osteoclasts, (Figure 4f). Importantly, the microscopy was corroborated by fluorescent SDS PAGE analyses seen in (Figure 4g). Cells treated with **GD32** resulted in specific CTSK labeling as demonstrated by a single fluorescent band at 25 kDa. Both **GD30** and ODN pretreatment depleted CTSK labeling above 25 kDa, (Figure 4g). Since **GD42** is a reversible probe, the probe‐enzyme complex is not stable in SDS PAGE conditions and could not be detected, as seen in (Figure 3). These results show that **GD32** is a selective CTSK irreversible probe that enables the detection of active mouse CTSK both by fluorescent microscopy and by fluorescent SDS PAGE. However, the reversibility of **GD42** only enables the detection of active CTSK using live fluorescent microscopy.

**Figure 5:**
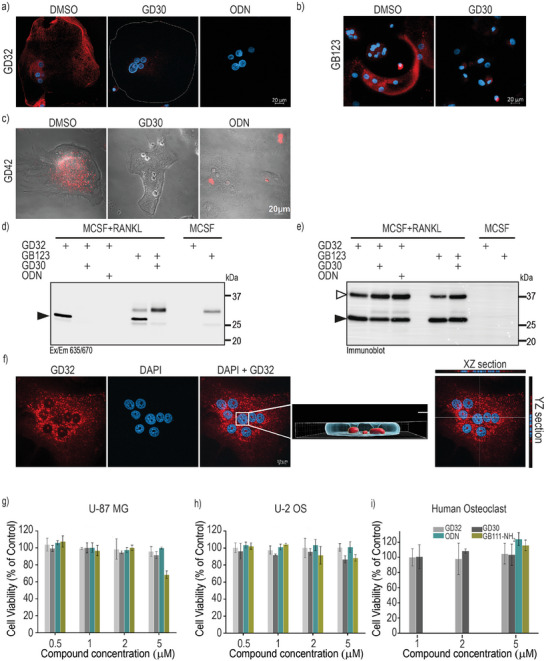
Labeling of endogenous cathepsin K in intact human osteoclast cells. Enriched human monocytes from PBMC were isolated from healthy donors’ blood, cells were treated with MCSF and RANKL or MCSF alone as indicated for 14 days. a–c) Osteoclasts were treated with vehicle (DMSO) or inhibitors **ODN** or **GD30** [3 µm] in a culture medium for 2 h, followed by incubation with **GD32** or **GD42** for 8 h or **GB123** for 2 h similar to as described in Figure 4. (a, b) Confocal fluorescent microscopy of **GD32** and **GB123** treated cells after fixation. c) Live cell imaging with **GD42**. d) SDS PAGE of the human osteoclasts and macrophages incubated with DMSO, **ODN** or **GD30** [5 µm] followed by incubation with **GD32** or **GD42** [5 µm] for 8 h. e) The same gel was analyzed by western blot with an anti‐CTSK antibody. The position of mature CTSK is indicated by a black arrowhead. f) The 3D analysis of the multinucleated osteoclast cells pretreated with **GD32** (1.25 µm) for 8 h. The XZ and YZ sections confirm the presence of CTSK activity within the cell's nucleolus. Red fluorescence marks cathepsin activity (ABPs) and blue fluorescence marks nucleus (DAPI). g–i) Cell viability assay. U‐2 OS, U‐87 MG and enriched monocytic cells were incubated with different concentrations of probe or inhibitor as indicated. After 48 h incubation cells were fixed, and viability was measured by methylene blue. The data were expressed as a percentage of control; data represent the mean of two experiments with triplicates for each treatment (± Standard Deviation).

We then tested the ability of **GD32** to selectively detect CTSK activity in human osteoclasts. Human peripheral blood mononuclear cells (PBMC) were collected from healthy donors, enriched with monocytes, and treated with MCSF and RANKL for 12–14 days to induce osteoclast maturation.^[^ ^53^ ^]^ The cells were then treated with **GD32**, lysed and analyzed by fluorescent SDS PAGE, and blotted with anti‐CTSK (Figure S8, Supporting Information). After 14‐day maturation, the differentiated giant multinucleated osteoclasts were treated with **GD32** for 8 h and visualized with fluorescent microscopy. The presence of Cy5 fluorescence (red) was observed in a vesicular pattern throughout the cytoplasm, with tiny puncta located at the margins of the cytoplasm in multinucleated cells. This distribution is consistent with the expected activity of CTSK, (**Figure** **5**a). To confirm the origin of the signaling intensity, osteoclasts were pretreated with the CTSK‐specific inhibitor **GD30**, or ODN prior to incubation with **GD32**. The inhibition of the signal due to the pre‐incubation further shows the specificity of the probe in human cells. While human osteoclasts are known to highly express CTSK, a detectable amount of CTSB also exists in these cells.^[^ ^54^ ^]^ Therefore, as a control, osteoclasts were treated with **GB123**, and analyzed by both microscopy and gel. Osteoclastic cells gave a strong fluorescence signal with **GB123** in the cytoplasm similar to **GD32,** although without specific binding to CTSK, (Figure 5b). The covalent reversible probe **GD42** detected active CTSK present only in live cells, while the signaling intensity was attenuated in the presence of both CTSK‐specific inhibitors **GD30**, and ODN, (Figure 5c). To verify these findings, we lysed the osteoclasts and macrophages treated with the various inhibitors followed by the incubation with **GD32** (**GB123** was used as a control) cells were analyzed by SDS‐PAGE, (Figure 5d). Macrophages were generated and used as control cells that have negligible CTSK.^[^ ^55^ ^]^ These data emphasize the selectivity and strongly labeling of CTSK by **GD32**, without interacting with the rest of the active cathepsins in the sample. In addition, the specificity of **GD30** is demonstrated again as it eliminates only the CTSK band leaving the remaining cathepsins active. The presence of active CTSK in human osteoclasts was confirmed by western blot of the same gels, (Figure 5e).

A thorough three‐dimensional examination by confocal microscopy was undertaken on human osteoclasts that were treated with **GD32** in order to examine the nuclear CTSK activity in these cells (Figure 5f). This is the first time that the activity of CTSK has been detected in the nucleus. The XZ and YZ sections (Figure 5f) revealed the presence of CTSK activity in the nucleolus. In summary, these findings clearly demonstrated that both **GD32** (covalent irreversible) and **GD42** (covalent reversible) are CTSK‐specific activity‐based probes (ABPs) capable of labeling both human and mouse CTSK.

To determine the cytotoxicity of our compounds, we followed the proliferation of U‐2 OS and U‐87 MG cell lines as well as primary human osteoclasts. No toxicity was noted by **GD30,** and **GD32** (Figure 5g–i), however, the pan cathepsin inhibitor **GB111‐NH_2_** exhibited cytotoxicity in the U‐87 MG cell line, likely because it inhibited the activity of several cysteine cathepsins.

**Figure 6:**
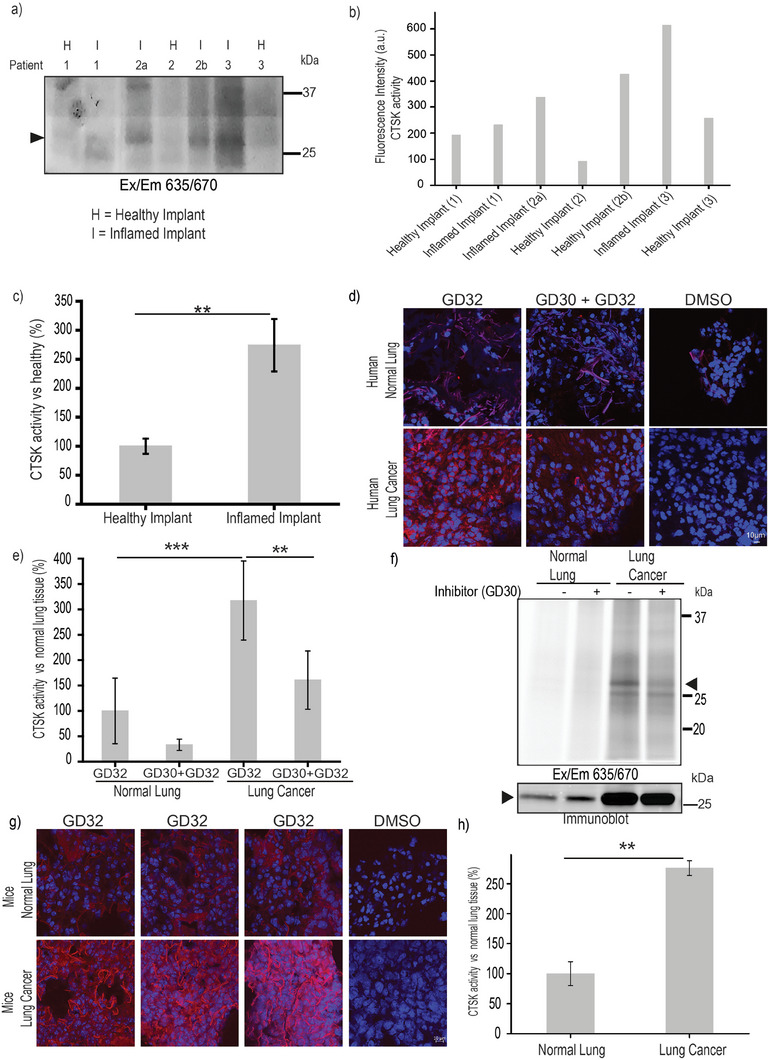
Human Dental and lung samples labeled by **GD32**. a) GCF fluid from healthy and inflamed teeth implants were treated with **GD32** for 6 h. Samples were then separated by SDS‐PAGE and scanned for fluorescence by a Typhoon scanner at 635/670 nm. b) Quantification of active CTSK in different patients and c) Average band intensity samples presented in a), values are the mean ± SEM. ^**^ *p* < 0.01; ^***^ *p* < 0.001. d) Normal and cancerous freshly resected human lung samples were treated with vehicle‐DMSO or **GD30** (5 µm) for 1 h and then incubated with **GD32** for 4 h. The samples were stained with DAPI (nucleus) and subjected to fluorescence microscopy. Representative images are shown. e) Quantifying fluorescence in lung samples imaged by confocal microscopy. The mean emission of **GD32** (Cy5 filter) intensity inside cells was quantified by NIS Elements software. The **GD32** signal was compared to the signal in normal tissue designated as 100% ± Standard error. f) Human lung samples were treated with DMSO vehicle or **GD30** (10 µm) for 2 h followed by the incubation with 5 µm **GD32** for 12 h, cells were lysed, and equal proteins were analyzed by fluorescent SDS‐PAGE. Anti CTSK western blot of the gel is shown below. g) Normal and cancerous mouse lung samples were treated with DMSO or **GD32** (0.75 µm) for 4 h. The samples were stained with DAPI (nucleus), and subjected to fluorescence microscopy as in d. h) Quantification of Cy5 fluorescence in different sections of normal and cancerous mouse lung samples. The mean emission of **GD32** (Cy5 filter) intensity inside cells was quantified by NIS Elements software, and divided by the total number of nuclei within each frame (marked by DAPI). The **GD32** signal was compared to the signal in normal tissue designated as 100%.

The newly developed chemical tool, **GD32**, was investigated for its utility in the early detection of CTSK‐related bone loss following dental implants. CTSK activity was analyzed in the gingival crevicular fluid (GCF) of patients with dental implants. The different samples include GCF from inflamed implants, healthy implants, and as controls, healthy teeth. GCFs were collected using filter strips and flash‐frozen. Protein extracts were then labeled with **GD32**.^[^ ^56^ ^]^ CTSK activity was detected by fluorescence SDS‐PAGE, the quantification reveals a 2.7‐fold (SEM = ± 0.2) increase in CTSK activity in inflamed implant samples relative to healthy implant samples and a 2.6‐fold (SEM = ± 0.6) increase relative to inflamed teeth samples (**Figure** **6**a–c; Figure S9, Supporting Information). These results show that reporting on CTSK activity may aid in the early detection of dental diseases and may suggest the use of CTSK inhibitors as an effective treatment to prevent implant loss in appropriate cases.

We further investigated the ability of **GD32** to detect CTSK activity in human lung cancer tissue. We applied our chemical tools to study the activity and localization of active CTSK in freshly resected human lung cancer and normal lung tissues. Although there are literature reports showing that normal lung tissues display a significant amount of CTSK protein, the activity is still unclear due to the lack of a proper tool.^[^ ^57^, ^58^ ^]^ In this context, we examined whether **GD32** can be used to visualize the presumably enhanced activity of CTSK in lung cancer. Freshly resected human lung tissues, normal and cancerous, were treated with **GD32** for 4 h, and CTSK activity was captured by fluorescent microscopy, (Figure 6d), and quantified in (Figure 6e). An enhanced fluorescent signal within the cells of the lung cancer tissues relative to normal lung tissues was shown. Importantly, this signal was eliminated by a specific CTSK inhibitor **GD30**.

We confirmed our above finding after lysing the tissues and visualizing the probe‐labeled protein by fluorescent SDS‐PAGE, (Figure 6f). The fluorescent band corresponds to active CTSK (marked) in tumor tissues which was blocked after pretreatment with the inhibitor. Moreover, the elevated CTSK activity in mice lung tumors compared to normal lung tissue was assessed by **GD32,** (Figure 6g). Confocal microscopic imaging demonstrates that the activity of the enzyme was 2.7 times higher in lung tumors compared to normal lung tissues, (Figure 6h). Interestingly, the probe distribution was higher within the cells of the lung cancer tissue and near the collagen fibers. These results demonstrate the utility of **GD32** to detect the localization and distribution of active CTSK in both human and mouse lung tissues by fluorescent microscopy.

## 3 Conclusion

Cysteine cathepsins have drawn attention in the pharmaceutical industry and academia due to their active involvement in numerous pathophysiological processes. Among the lysosomal cysteine proteases, CTSK became an attractive target for antiresorptive drug targets as its levels have been shown to be significant in the disease prognosis. Besides bone resorption, this enzyme has far‐reaching action within other organs. Hence, it is crucial and of great importance to create innovative tools to study CTSK activity in both healthy and diseased states to tackle essential inquiries regarding its physiological functions.

While using ABPs for selective enzyme imaging is of great value, identifying probes for a specific protease is difficult due to enzymes with similar structures and almost identical substrate specificity. With the help of our advanced synthetic knowledge in the design and evaluation of ABPs, we created two ABPs specific for CTSK on the basis of their modes of action. Of these, a covalent irreversible probe, **GD32,** was applied for selective, sensitive, and quantitative assessment of CTSK in cells as well as in human tooth implants. Identification of CTSK activity in periodontics can indicate suitable treatment and as a result prevention of implant failure.

The selective CTSK imaging potential of **GD32** was successfully demonstrated in both cancer cells and primary human and mice osteoclasts. We also show active CTSK in the nucleus, specifically in the nucleolus, of the multinucleated osteoclast. These data align with previous reports that show the expression of CTSK and C‐FOS in the nucleus of multinuclear osteoclasts.^[^ ^59^ ^]^ Active nuclear CTSK has never been reported before, emphasizing the importance and necessity of the here‐developed chemical tools that enable high‐quality microscopy. The power of this selective tool was also demonstrated in human and murine lung tumor tissues, detecting the higher activity of CTSK compared with healthy lung tissue. Moreover, the covalent irreversible probe **GD32** was utilized to investigate the effect of pH on the maturation of CTSK.

The reversible binding mechanism of **GD42** could be used for providing the exact localization of CTSK in the subcellular compartments of primary and cancer cells with the help of live cell imaging techniques. In addition, an enlarged potential safety and reduced immunogenicity risk is associated with the non‐permanent modification of a protein resulting from the probe's reversible covalent mechanism.

Furthermore, our findings explain the significant challenge faced by the scientific community in detecting CTSK activity in cell lysate. We show that Cystatin B, a potent CTS endogenous inhibitor, binds CTSK when cells are lysed and limits its activity, while in intact cells, this interaction is negligible (Figure 2h). These data indicate the correct way to detect CTSK activity and elucidate the challenges encountered in CTSK‐activity‐related research throughout the years.

Taken together, we successfully developed specific CTSK activity‐based probes that were used to detect CTSK activity in disease states regardless of the presence of other cathepsin activity. We believe that the unique selectivity and sensitivity of these probes toward CTSK, will allow future applications of detecting the on and off‐target activity of CTSK inhibitors in future drug development. These probes may also be suitable tools for investigating the exact in vivo function of the enzyme in different organs of murine models.

## Conflict of Interest

The authors declare no conflict of interest.

## Author Contributions

G.B. and G.D. performed conceptualization. G.D., E.M., and D.T. performed methodology. G.D., E.Y., R.S., E.M., and J.L. performed investigation. O.W., Y.H.H., D.P., N.S., and S.Y. provided materials. G.D., E.M., and G.B. wrote the original draft. J.L. and S.Y. reviewed and edited the final manuscript. G.B. supervsed the research.

## Supporting information

Supporting Information

## Data Availability

Research data are not shared.
