# Supplementary material for: Development and Application of Reversible and Irreversible Covalent Probes for Human and Mouse Cathepsin‐K Activity Detection, Revealing Nuclear Activity
Source: Adv Sci (Weinh). 2024 Jul 5;11(38):2401518. doi: 10.1002/advs.202401518 (PMC11481179; doi:10.1002/advs.202401518)
Supplement: Supplementary file 1 — Supporting Information [file ADVS-11-2401518-s001.pdf]

ADVANCED SCIENCE
Open Access

Supporting Information

for *Adv. Sci.*, DOI 10.1002/advs.202401518

Development and Application of Reversible and Irreversible Covalent Probes for Human and Mouse Cathepsin-K Activity Detection, Revealing Nuclear Activity

*Gourab Dey, Reut Sinai-Turyansky, Evalyn Yakobovich, Emmanuelle Merquiol, Jure Loboda, Nikhila Sridharan, Yael Houri-Haddad, David Polak, Simon Yona, Dusan Turk, Ori Wald and Galia Blum**

Supporting Information

Development and Application of Reversible and Irreversible Covalent Cathepsin K Activity-Based Probes in a Variety of Human and Mice Samples, Revealing Nuclear Activity

Gourab Dey,[1] Reut Sinai-Turyansky,[1] Evalyn Yakobovich,[1] Emmanuelle Merquiol,[1] Jure Loboda,[2] Nikhila Sridharan,[3] Yael Houri Haddad,[4] David Polak,[4] Simon Yona,[3] Dusan Turk,[2] Ori Wald,[5] and Galia Blum*[1]

[1] The Institute of Drug Research, The School of Pharmacy, The Faculty of Medicine, The Hebrew University of Jerusalem, Jerusalem, Israel.

[2] Department of Biochemistry, Molecular and Structural Biology, J. Stefan Institute, Ljubljana, Slovenia.

[3] The Institute of Biomedical and Oral Research, The Faculty of Dental Medicine, The Hebrew University of Jerusalem, Jerusalem, Israel.

[4] Department of Prosthodontics, The Faculty of Dental Medicine, The Hebrew University of Jerusalem, Jerusalem, Israel

[5] Department of Cardiothoracic Surgery, Hadassah Hebrew University Medical Center, The Faculty of Medicine, The Hebrew University of Jerusalem, Jerusalem, Israel.

Supporting Figures

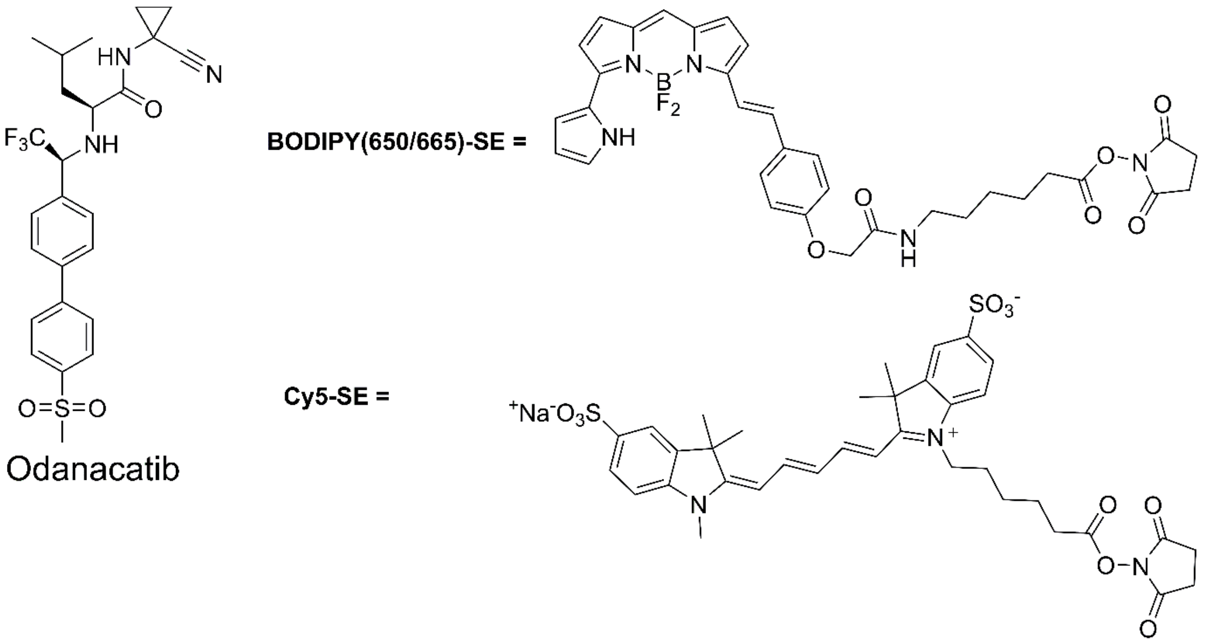

Figure S1. Chemical structure of: Odanacatib, Cy5-SE, and BODIPY-SE 650/665, Succinimidyl Ester (SE).

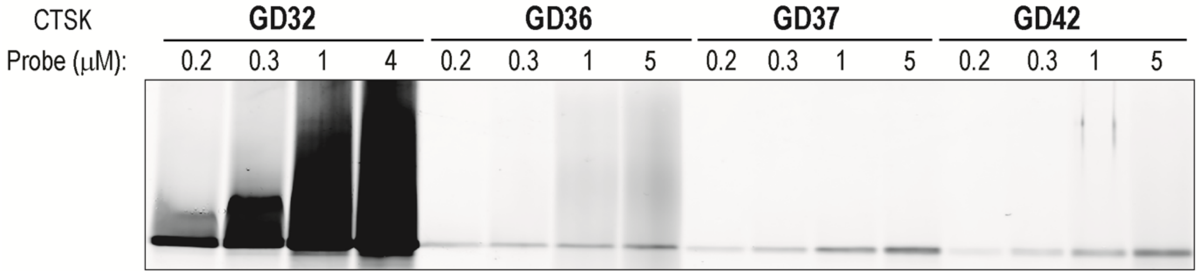

Figure S2. Recombinant cathepsins [0.5 μM] were treated with increasing concentrations of ABPs for 1 h. Samples were separated by SDS-PAGE and scanned for fluorescence by a Typhoon scanner at 635/670 nm.

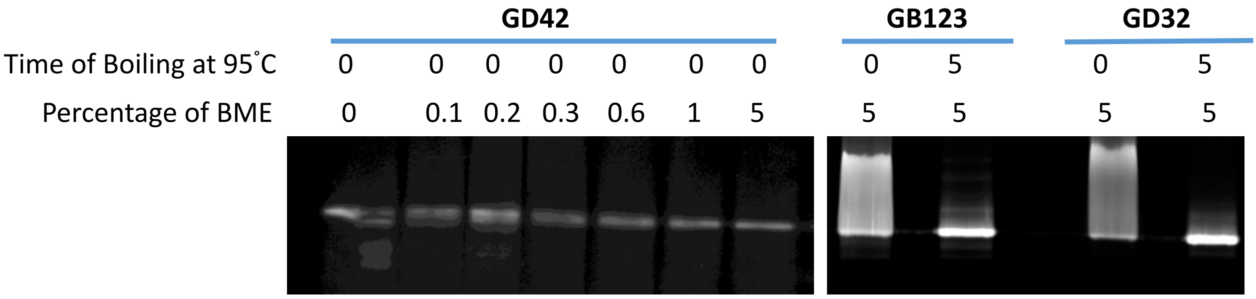

Figure S3. Recombinant CTSK (0.5 μM) were treated with **GD42**, **GD32** (5 μM) or **GB123** (1 μM) for 1 h. After that enzyme-**GD42** complexes were treated with different concentrations of beta-mercaptoethanol (BME) in the sample buffer. **GD32** and **GB123** probe-enzyme complexes were treated with a sample buffer containing 5% beta-mercaptoethanol. Samples were separated on SDS-PAGE and scanned for fluorescence by a Typhoon scanner at 635/670 nm.

a)

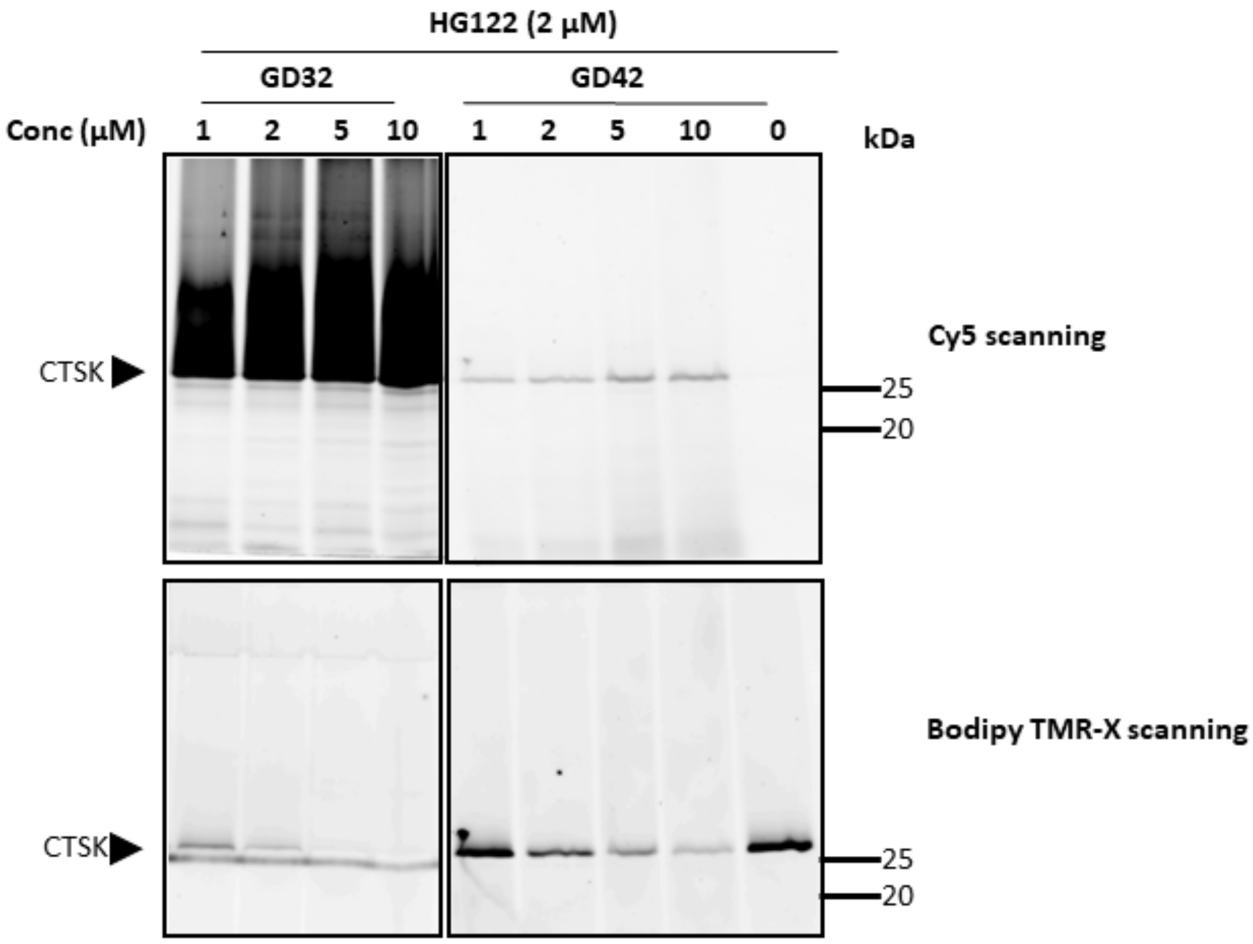
HG122 (2 µM)
GD32
GD42
Conc (µM) 1 2 5 10 1 2 5 10 0
kDa
CTSK
Cy5 scanning
25
20
CTSK
Bodipy TMR-X scanning
25
20

b)

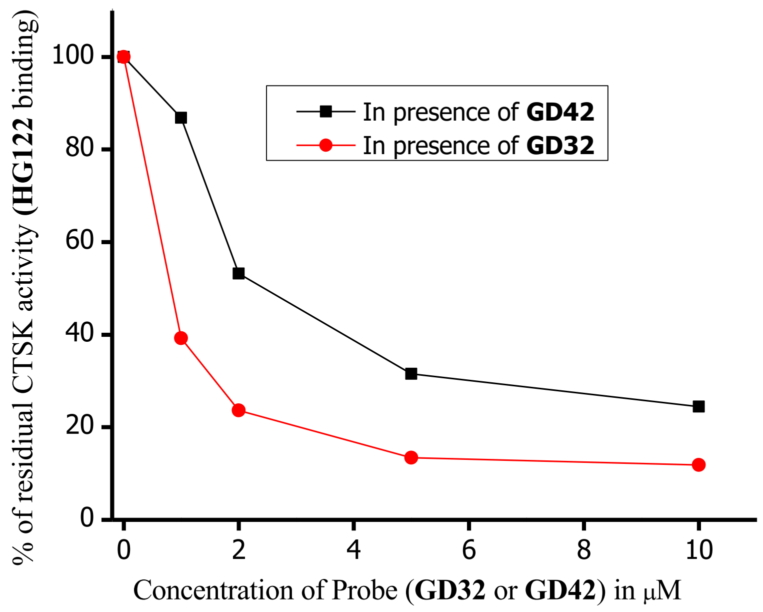
% of residual CTSK activity (HG122 binding)
100
80
60
40
20
0
0 2 4 6 8 10
Concentration of Probe (GD32 or GD42) in µM
In presence of GD42
In presence of GD32

Figure S4. (a) Recombinant CTSK (0.5 μM) was incubated with the indicated concentration of **GD42**, **GD32** or DMSO for 1 h at 37° C. Then, **HG122** (2 μM), a BODIPY TMR-X labeled pan-cathepsin probe was added and incubated for another hour at 37° C. Proteins were analyzed by fluorescent SDS PAGE scanning by a Typhoon flatbed scanner for Cy5, and BODIPY TMR-X (Cy3) labeling residual CTSK inhibition. (b) Quantification of CTSK labeling by **HG122** (2 μM) (Cy3 signal) after treatment with increasing concentration of **GD32** or **GD42.** The percentage is relative to the maximum binding of **HG122** (Cy3 signal) with vehicle pretreatment determined as 100% activity. Increasing concentration of **GD32** or **GD42** decreased **HG122** binding, indicating CTSK inhibition. The irreversible nature of **GD32** results in better inhibition relative to **GD42** (reversible). The X-axis denotes the concentration of **GD32** and **GD42** in μM, Y-axis is the percent of residual CTS activity, **HG122** binding as detected by the intensity of the Cy3 signal.

a)

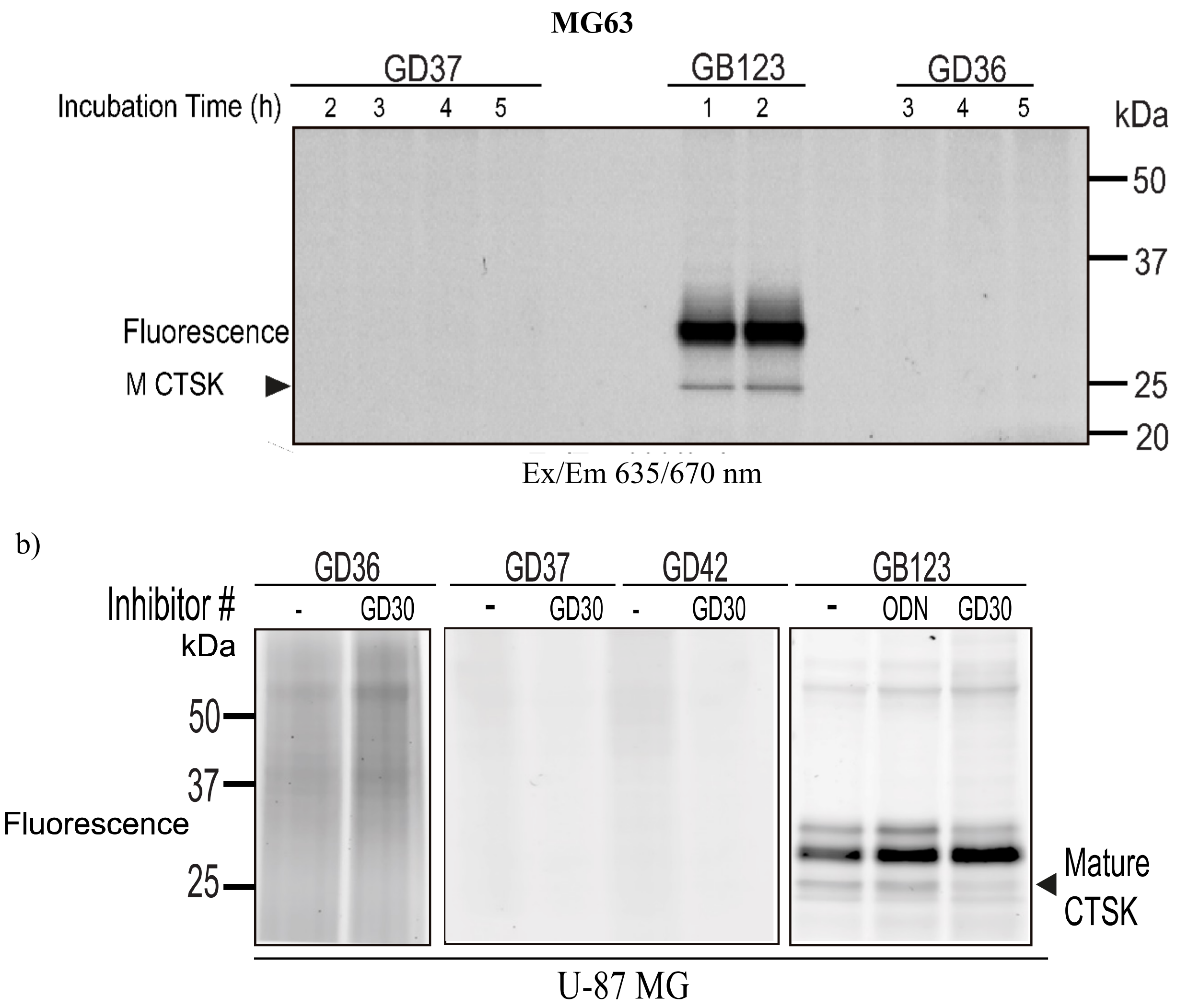

Figure S5. Direct labeling of endogenous CTSK in intact (a) MG-63, (b) U-87 MG. (a) MG-63 cells were treated with **GD37, GD36**, and **GB123** (5 µM) in a culture medium for indicated durations. (b) U-87 MG cells were treated with **GD36, GD37, GD42, or GB123** (5 µM) in the presence and absence of CTSK specific inhibitor **GD30** or **Odanacatib** (5 µM) for 2 h in a culture medium, cell lysates were analyzed as described in **Figure S2**.

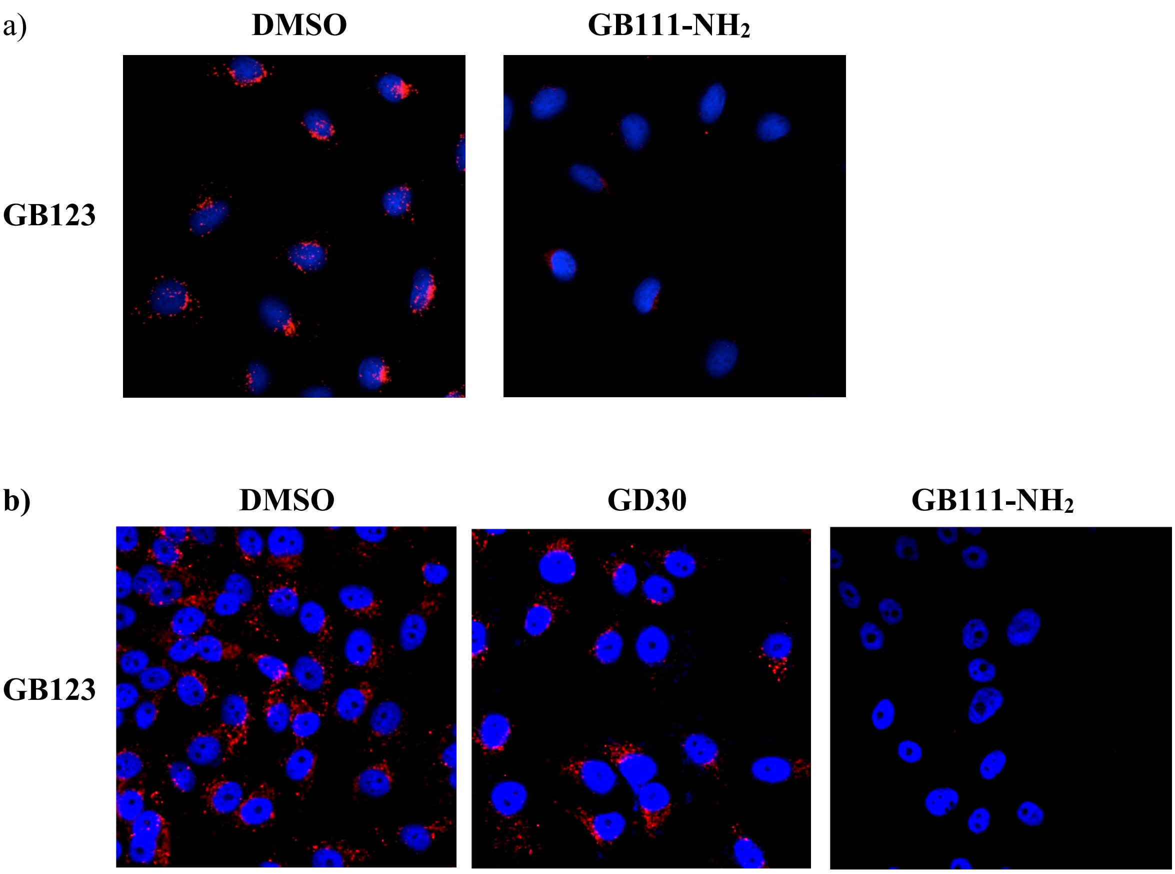

Figure S6. Cultures of a) U2-OS, and b) MG-63 cells were grown in 8-well chambers. Cells were either pre-treated with the pan cathepsin inhibitor **GB111-NH$_2$** (3 µM) or CTSK-specific inhibitor **GD30** (3 µM) or with DMSO (0.1%) for 2 h and labeled by the addition of **GB123** (1.25 µM) to growth media for 2 h, after fixation cells were stained with DAPI and imaged by using confocal fluorescence microscopy with Cy5 filter.

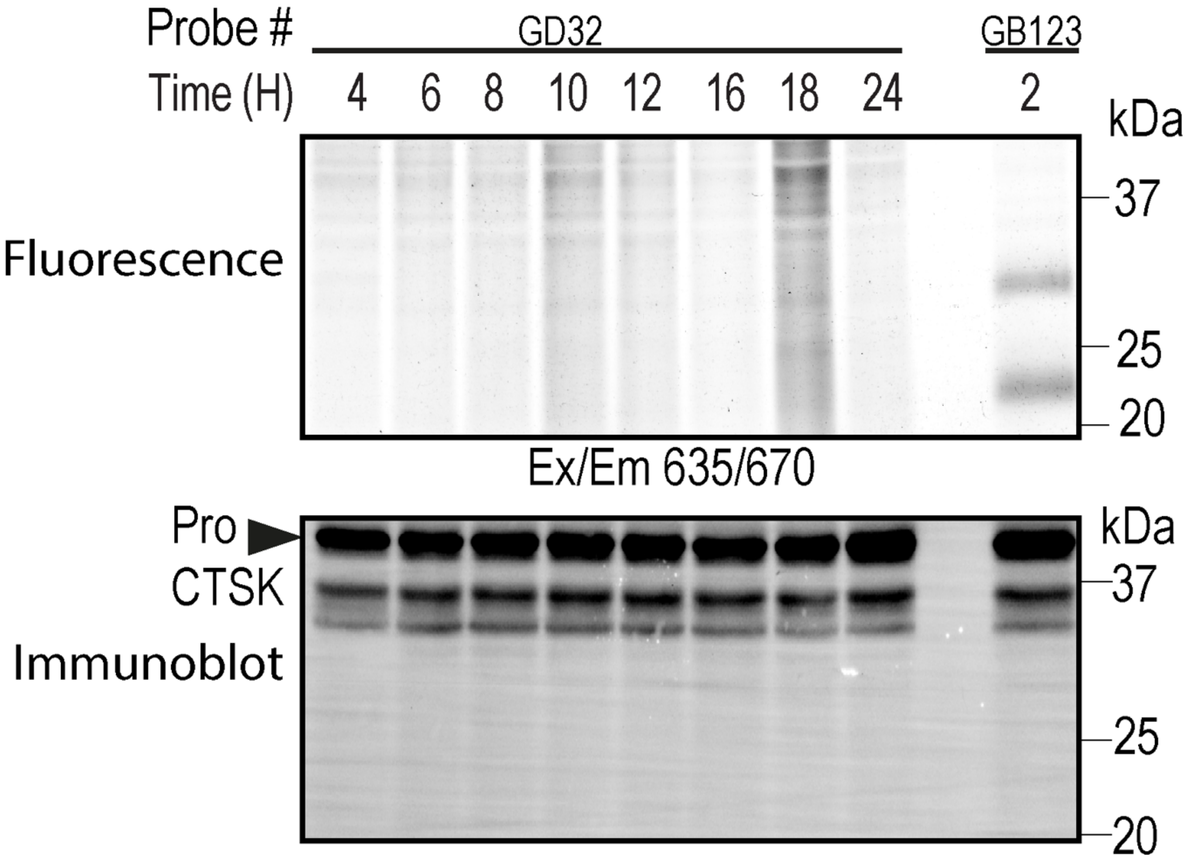

Figure S7. Cathepsin labeling in intact Jurkat cells that lack mature CTSK. Jurkat cells were treated with **GD32** or **GB123** (5 µM) in a culture medium for different durations up to 24 h, followed by cell lysis, and fluorescent SDS-PAGE analysis. No fluorescent bands were detected after labeling with **GD32** as these cells lack mature CTSK. The gel was analyzed by western blot with anti CTSK antibody. While pro-CTSK is detected, indicated by the black arrow, the mature CTSK is not detected.

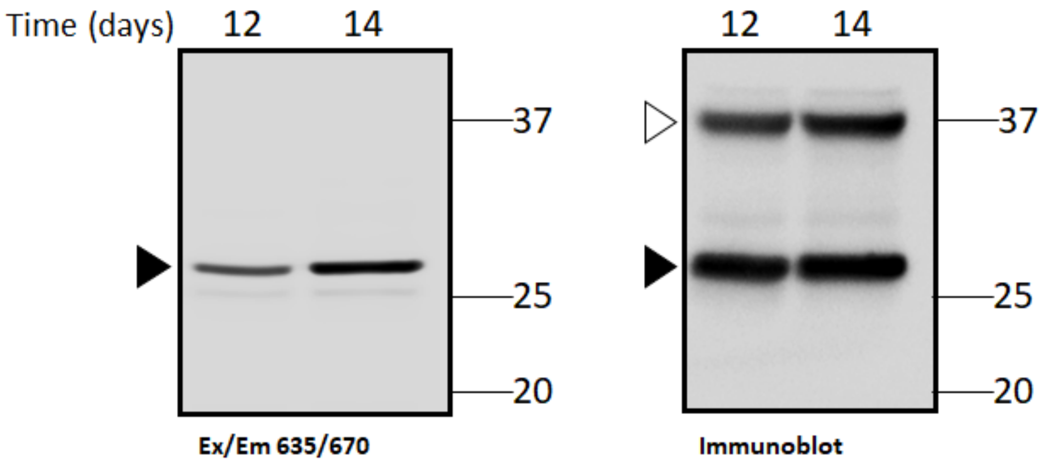

Legend: ▷ pro-CTSK ► mature CTSK

Figure S8. Human Osteoclasts labeled with **GD32**. Purified monocytes from human PBMC were treated for 12 or 14 days with RANKL and MCSF and then labeled with **GD32** (5 µM). Lysates were separated on a 12.5% SDS PAGE and scanned for Cy5 (on the left). The gel was transferred and immunoblotted with a CTSK antibody (on the right).

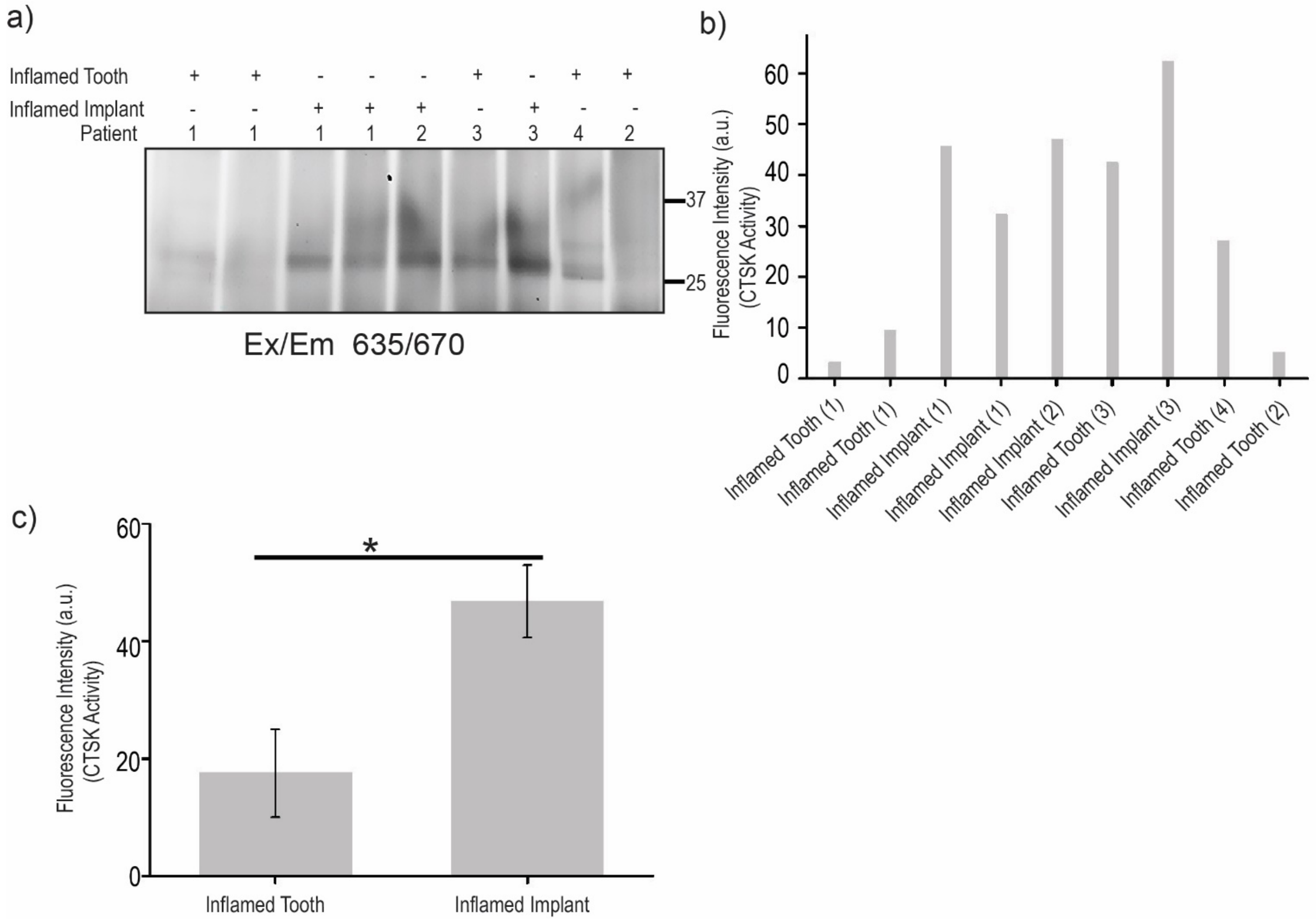

Figure S9. The samples of inflamed implants and teeth were labeled with **GD32** for a duration of 6 h. The samples were analyzed as described in **Figure S2**. b) Quantification of the CTSK band intensity in each sample. C) Average of the quantification of samples. Values are the mean ± SEM. *P < 0.05; **P < 0.01; ***P < 0.001.

General Methods

The solvents (*n*-hexane, dichloromethane (DCM), methanol, and ethyl acetate) which were used for purifying compounds through column chromatography, were purchased from a commercial source and used without distillation. All resins and reagents were purchased from commercial suppliers and used without further purification. In column chromatography, silica gel (120-200 μm,60 Å) was used as the stationary phase. Merck silica gel 60-F-254 plates were

used for TLC. Phosphate Buffer Saline (PBS) was purchased from Biological Industries (pH = 7.4) and was used as a buffer system in all experiments if not indicated otherwise. The stock solution of compounds (50 mM) was prepared in DMSO, and the DMSO final concentration in cell experiments did not exceed 0.2% DMSO in the medium. All water-sensitive reactions were performed in anhydrous solvents under a positive pressure of argon. Cy5, and BODIPY succinimidyl ester (SE) was purchased from Lumiprobe. Reactions were characterized by liquid chromatography-mass spectrometry (LC-MS) (Thermo Scientific LCQ Fleet attached to an Accela UPLC system) using reverse-phase chromatography with a water/acetonitrile gradient. All final compounds were purified by C18 reverse phase HPLC in acetonitrile/water gradient. Recombinant human CTSB, CTSK, CTSL, and CTSS were supplied by B. Turk (J. Stefan Institute).[1] The animal ethics committee of the Hebrew University approved all animal procedures, approval # MD-21-16535-4. Human blood, dental, and lung samples were taken in accordance with the Declaration of Helsinki (approval number for blood samples: HMO-0268-19, dental samples: 0674-16-HMO, and lung samples: 0235-21-HMO)

Instruments and measurements: Bruker Advance III 500 MHz spectrometer was used for measuring ^{1}H and ^{13}C NMR spectra of the compounds in $CDCl_3$, CD_3OD, and DMSO-d_6. Fluorescent gels were scanned by a Typhoon FLA 9500 flatbed laser scanner (GE Healthcare Bio-Sciences AB, Uppsala, Sweden). BioRad ChemiDox XRS+ was used for western blot analysis. Olympus FV10i inverted microscope (Tokyo,Japan) with Fluoview software or Zeiss Confocal LSM 980 with Airyscan (Germany) with Zen software were used for confocal measurement.

In vitro kinetic evaluation of labeling recombinant cathepsins:

Reactions were monitored for 30 min at 37° C in 96-well black flat-bottom microplates (Greiner, Germany) using Tecan INFINITE M1000 pro plate reader (Tecan, Switzerland), substrates Z-RR-AMC (CTSL) or Z-FR-AMC (CTSS, CTSB and CTSK), and excitation and

emission wavelengths of 370 and 460 nm, respectively. Cathepsin concentrations in the assay were 0.4 nM (CTSK) or 5 nM (CTSS, CTSB, and CTSL). The buffer was 100 mM Na_2HPO_4/NaH_2PO_4, 50 mM NaCl, 5 mM DTT, 0,1% Peg-6000, pH 6. For irreversible inhibitors, reaction data were fitted to the one-phase association formula in GraphPad Prism 9 software: $Y=Y_0$ + (Plateau-Y_0)*(1-exp(-K*X)) where in this case Y_0 and Y represent fluorescence signal at times 0 and *t*, respectively, K represent the observed reaction rate k_{obs} and X the inhibitor concentration. Inactivation rates at each inhibitor concentration were obtained by subtracting the inactivation observed in the control sample: $k_{obs} - k_{ctrl}$. The maximal rate of inactivation (k_{inact}) was obtained as the asymptote from equation $Y = k_{inact}*X/(K_i^{app} + X)$ where X is inhibitor concentration, Y is the observed inactivation rate for each X, and K_i^{app} is the apparent inhibition constant. K_i values were calculated from IC_{50} or from K_i^{app} using formulas $K_i = K_i^{app} / (1 + K_m/S)$ and $IC_{50} = E_0/2 + K_i^{app}$, where K_m stands for Michalis-Menten constant, S for substrate concentration and E_0 for cathepsin concentration. The highest inhibitor concentrations in the assay were 50 µM. Measurements were performed in several independent duplicates. Average values are presented with standard deviations.

Recombinant cathepsin inhibition: Recombinant human CTSL, CTSB, CTSK, and CTSS (0.5 µM) were pretreated with different concentrations of inhibitors (**GD30**, ODN) for 1 h in reaction buffer (50 mM acetate, 4 mM DTT and 5 mM $MgCl_2$, pH 5.5) at 37° C. **GB123,** 1 µM, was added, and incubated with samples for 1 h at 37° C. The reaction was stopped by the addition of sample buffer 4x (40% glycerol, 0.2 M Tris/HCl pH 6.8, 20% beta-mercaptoethanol, 12% SDS, and 0.4 mg/ml bromophenol blue). Samples were then boiled at 95° C for 2 mins, separated on a 12.5% SDS gel, and scanned for fluorescence by a Typhoon scanner FLA 9500 at excitation/emission wavelengths of 635/670 nm.

Recombinant cathepsin labeling: Recombinant human CTSL, CTSB, CTSK, and CTSS (0.5 µM) were pretreated with different concentrations of probes (**GD32**, **GD36**, **GD37**, or **GD42**)

for 1 h in reaction buffer (50 mM acetate, 4 mM DTT and 5 mM $MgCl_2$, pH 5.5) at 37° C. The reaction was stopped and samples were analyzed as described above.

Mass Spectrometry:

Recombinant CTSK (1.5 μM) was incubated with DMSO or **GD32** (15 μM) in buffer acetate for 5 h at 37° C. The mixtures were then freeze-dried, and acetonitrile was added to remove the salt from buffer. Samples were prepared by adding an equal volume of Trans-3,5-dimethoxy-4-hydroxy cinnamic acid as a matrix. Matrix-assisted laser desorption ionization time-of-flight (MALDI-TOF) mass spectrometry was performed on a BRUKER microflex LRF MALDI-TOF/TOF. Spectra were acquired using the Bruker Daltonics Flex Control 3.4 and analyzed using Bruker Daltonics Flex Analysis 3.4.

Cell culture: Human osteosarcoma cells (MG-63), human glioblastoma cell line (U-87 MG), and human lung carcinoma cells (A549) were cultured in Dulbecco's Modified Eagle's Medium (DMEM) medium containing 10% fetal bovine serum (FBS), 1% penicillin, and 1% streptomycin. Human bone osteosarcoma epithelial cells (U-2 OS) were cultured in McCoy's 5A Medium containing 10% FBS, 1% penicillin, and 1% streptomycin. Jurkat cells were cultured in RPMI medium containing 10% FBS, 1% penicillin, and 1% streptomycin.

Mice osteoclast were prepared by extraction of bone marrow from the tibia and fibula of BALB/C mice, Cells were cultured in alpha MEM medium containing 10% FBS, 1% penicillin, and 1% streptomycin with 30 ng/ml RANKL and 20 ng/ml MCSF for 7-9 days until the multinucleated giant osteoclast cells have appeared as previously described [2]. For the macrophage preparation, the same procedure was maintained but without RANKL. All cells were maintained in an incubator at 37^0C with a 5% CO_2/air environment. Cell lines were kind gifts from the laboratories of Dr. Alex Rouvinski (Hebrew University), Prof. Ronit Satchi-Fainaro (Tel Aviv University), and Prof. Dina Ben Yehuda (Hadassah Hospital). All cells were negative for mycoplasma.

Evaluation of the permeability of GD30, and ODN to intact cells, competition assay: MG63, U87-MG, A549, and U-2 OS cells ($5x10^5$ cells/well) were seeded in a six-well plate one day before treatment. Cells were treated with vehicle or different concentrations of inhibitors **GD30**, **GB111-NH$_2$** or ODN (5 μM) that were pre-dissolved in 0.1% DMSO in a culture medium. After 2 h of inhibitor incubation, residual cathepsin activity was labeled with **GD32, GD42 or GB123** (5 μM).[3] Cells were washed with PBS and lysed with the addition of RIPA buffer, pH 7.4 (1% NP-40 (nonyl phenoxypolyethoxylethanol) 0.1% SDS, 0.5% sodium deoxycholate). After adding sample buffer, samples were boiled for 10 minutes, and analyzed as described above.

Evaluation of probes in intact cells, direct labeling assay: MG63, U87-MG, or U-2 OS cells ($5x10^5$ cells/well) were seeded in a six-well plate one day before treatment. Cells were treated with inhibitors or with the vehicle in a culture medium at pH 7.4 for different incubation times followed by the addition of probes and incubated for different time in the same condition. Cell lysates were analyzed as described above. Proteins were then transferred to a PVDF membrane (Bio-Rad) and incubated overnight at 4° C with primary antibody diluted in TBS-T and 5% milk. For immunoblots, the following antibody was used: CTSK (Santa Cruz Biotechnology, catalog no - sc-48353, Lot no - J1122, clone - E-7) with 1:300 dilution. Membranes were then probed with Anti-mouse IgG, HRP-linked Antibody (Cell Signalling Technology, catalog no - CST-7076S, Lot no - 38) with 1:2000 dilution at room temperature for another hour, and a chemiluminescent signal was generated using the ECL kit (Millipore, WBLUR0500). Blot images were taken by ChemiDoc XRS camera (Bio-Rad) and densitometric analyses were carried out using ImageJ software (National Institute of Health).

Measurement of overlap coefficient

The colocalization (overlap coefficient) of the ABP (red) with immunoreactive CTSK (green) in U2-OS cells was calculated using Image J software. The JACoP plugin of Image J software generates pixel-intensity scatterplots to visualize localization patterns and measure the

colocalization (whole image) of CTSK (http://rsbweb.nih.gov/ij/plugins/track/jacop.html). The overlap coefficient was calculated based on randomly chosen three independent fluorescent images.

Co-Immunoprecipitation with CTSK antibody

Prior to labeling, a total of $15*10^5$ U87-MG cells were seeded in two 10 cm plates. One plate was used as a control, while the other plate was designated for labeling with **GD32.** The cells were exposed to 5µM **GD3**2 or **DMSO** for a duration of 2 h at 37° C. Cell lysates were obtained in the manner previously described. Protein quantification was performed. Samples of 50 µg were stored and subsequently used as input. The lysates were supplemented with CTSK antibody (Santa Cruz Biotechnology, catalogue no - sc-48353, Lot no - J1122, clone - E-7), 2µg/500 µg proteins and incubated overnight at 4° C with rotation. The following day, agarose beads 35-40µl of Protein A/G (Santa Cruz, sc-2003) were added to the lysate and left to agitate for 2 h. The beads were separated, then sample buffer was added to beads and supernatants, and samples were boiled for 10 min. The samples, including input, output, and immunoprecipitate, were separated using a 14% SDS PAGE gel. Afterward, an immunoblot was performed using an antibody specific to Cystatin B (Santa Cruz Biotechnology, Catalogue no - sc-166561, Lot no - D 0320, clone - F-5) with 1:500 dilution.

Labeling of mice osteoclast. Mice bone marrow cells ($5x10^6$ cells/well) were seeded in a six-well plate and treated for 6 days with RANKL and MCSF.[2] On the last day, cells were treated with **GD30** or ODN (5 µM) in a culture medium for 2 h, before being treated with **GD32** for 6 h or **GB123** for 2 h. Cell lysates were analyzed as previously described.

Evaluation of probes and inhibitors in human osteoclast sample:

Monocyte isolation from blood: Blood was taken in an aseptic non-touch technique, from consented human volunteers that were bled from the median cubital vein using a 21G needle, in accordance with the Declaration of Helsinki. Blood was collected in lithium heparin treated

vacutainers and RosetteSep® was added to enrich for monocytes followed by density centrifugation with Ficoll®Paque Plus density gradient media. The monocytes were then washed twice and resuspended in PBS, 2mM EDTA supplemented with 10% FCS and counted. $3x10^6$ cells were seeded per well in 24-well plates (Primaria, Corning®) in 1 mL of RPMI 1640 medium supplemented with 10% FBS and 50μg/mL gentamicin. The next day, the medium was removed, and the adherent cells were washed with 1 α-MEM and cultured for 14 consecutive days in α-MEM supplemented with 10% FBS 1% penicillin, and 1% streptomycin. (Biological Industries), 100 ng/mL RANKL (R&D Systems) and 25 ng/mL MCSF (R&D Systems) (for osteoclast) or with 25 ng/mL MCSF only (macrophage). Every 3 days the media was changed with the required amount of MCSF and RANKL or with MCSF only until the giant osteoclasts appeared, after 12 or 14 days of treatment. On the last day of treatment the inhibitor **GD30**, ODN or **GB111-NH$_2$** or vehicle (5μM) were added to the media and incubated for 2 h at 37° C. Then **5**μM **GD32** or **GB123** were added for 8 or 2 h (respectively) at 37° C. Cells were then washed with PBS and lysed in RIPA buffer. Cell lysates were analyzed as previously described.

Evaluation of probes in dental sample: Specialized paper strips were placed to absorb the dental gingival crevicular fluid (CGF) from patients, with Helsinki approval, and strips were frozen in liquid nitrogen. After which each sample was eluted in 50 μl cold RIPA and centrifuged at 300 rpm for 60 mins and then an additional 2 mins at 12000 rpm. The protein content of each sample was evaluated using BCA standard methods. An equal protein quantity (45 μg) was mixed with 5μM **GD32** and incubated at 37° C for 5 h. Sample buffer was added and samples were analyzed using fluorescent SDS-PAGE.

CTSK activity in different pH: A549 and U-2 OS cells ($5x10^5$ cells/well) were cultured in different pH of cultured medium for 1 day. The pH of the cultured medium was adjusted externally. Then 5 μM **GD32** was incubated with the cells at 37° C for 24 h. A549 cells were

incubated with increasing concentrations of **GD30** for 2 h at pH = 5, followed by the incubation with 5µM **GD32** for 24 h prior to the cell lysis. Cell lysates were prepared and analyzed as previously described.

Evaluation of probes and inhibitors in lung samples: Normal and tumor lung tissue was collected under Helsinki approval. Fresh lung samples were cut into small pieces and the tissues were minced and incubated in a 24-well plate in 1ml DMEM (with 10% FBS, P/S, and Glu). Normal and tumor samples were incubated in the same plate in DMEM with DMSO or 10µM **GD30** for 3 h. Then 5 µM **GD32** was added to all samples for an additional 12 h. At the end of the incubation, samples were centrifuged at 14000 rpm for 5 minutes, the supernatants were collected and the tissues were washed with PBS. Two volumes of cold RIPA were added to the tissue, and proteins were extracted using a bead homogenizer (Bullet Blender Storm - BBY24M, Next Advance) at speed 10 for 4 min at 4° C. Then lysates were prepared and analyzed as previously described.

Fluorescent imaging of CTSK activity in live cells: Cells were seeded in 8 well chambers, then treated with inhibitor or with vehicle for 1 h. Cells were labeled with the different probes as indicated. For live cell imaging, cells were imaged directly after treatment and three quick washes with PBS, with a confocal microscope equipped with a Cy5 filter. For fixed cells, cells were washed with PBS, fixed with cold methanol, and then cells were mounted with DAPI-Fluoromount-G® (SouthernBiotech, 0100-20) before visualization by confocal microscopy.

Cell Viability Assay:

U-2 OS, U-87 MG, and enriched monocyte cells were grown at 37^0 C with 5% of CO_2 in the respective medium as described previously. Cells were seeded (1500 cells/well in 96 wells plate) one day before the incubation with indicated probe or inhibitor at various concentrations. After 48 h cells were fixed, and viability was measured by quantitative colorimetric assay using

methylene blue according to the procedure reported by Ben-Bassat et al. [4] The data were expressed as a percentage of control; data represent the mean of 2 experiments with triplicates for each treatment (± standard deviation).

Fluorescent imaging of CTSK activity in lung tissue: Freshly resected human lung cancer and normal lung tissues or mouse normal lung and primary lung cancer were embedded in OCT, frozen in liquid nitrogen, and kept at - 80° C. Tissues were sectioned into 10 µm thick slices using a cryostat (Leica). The slides were defrosted and washed with 1X PBS. Then the slides were pretreated with DMSO or **GD30** (5 µM) in buffer acetate for 1 h at 37° C. Afterward, **GD32** (0.75uM) or DMSO in buffer acetate were applied to the slides for 4 h at 37° C. Three short washes with 1X PBS were done and the slides were left in 1X PBS for overnight incubation at 4° C. The next day, slides were mounted with DAPI-Fluoromount-G® before visualization by confocal microscopy. Olympus FV10i was used for human samples, whereas Zeiss was used for mouse samples.

Details of the synthesis of the compounds

Synthesis of Compound (2)

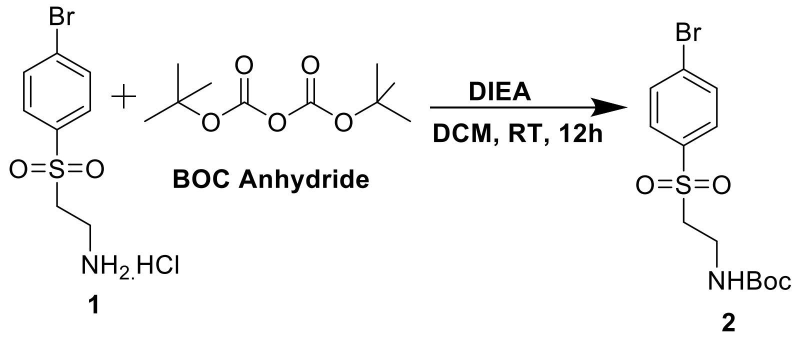

Commercially available compound **1** (292 mg, 0.97 mmol, 1 equivalent) and di-*tert*-butyl decarbonate (4 equivalent) were put in a round-bottom flask in a nitrogen atmosphere followed by the addition of dry DCM and 5 equivalents of DIEA. The reaction mixture was allowed to stir at room temperature for 12 h and the progress of the reaction was monitored by TLC. Once the reaction was complete, the mixture was washed with (1 N) HCL solution, separated by organic layer separation, and dried over sodium sulfate before concentrating by a rotary evaporator (rotavap). Finally, the compound was purified through column chromatography using a Hexane–Ethyl acetate system (320 mg, 90 % yield).

Synthesis of Compound (3)

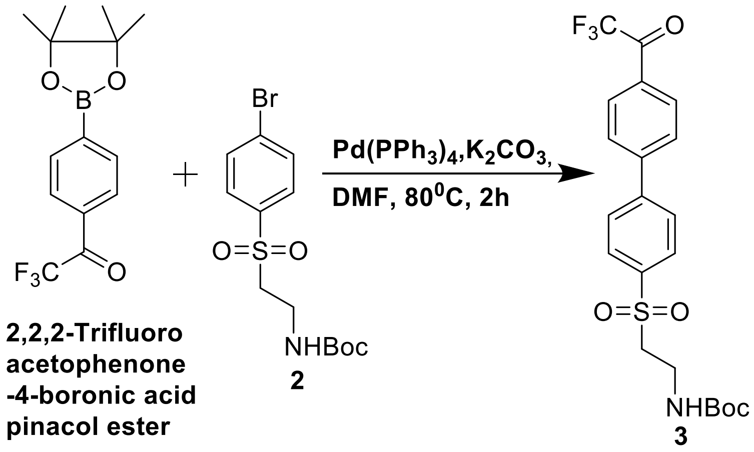

Commercially available compound **2,2,2-Trifluoro acetophenone-4-boronic acid pinacol ester** (269 mg, 0.896 mmol, 1 equivalent) and compound **2** (1.25 equivalent) were put in a round-bottom flask in a nitrogen atmosphere followed by the addition of dry DMF, 5 mol% of palladium tetrakis and (2 M) aqueous solution of potassium carbonate. The reaction mixture was allowed to reflux at 82^0 C for 2 h and the progress of the reaction was monitored by TLC. Once the reaction was completed, the solvent was evaporated using a rotavap. Then, the reaction mixture was dissolved in ethyl acetate before washing with (1 N) HCL solution, followed by separation of the organic layer that was dried over sodium sulfate before

concentrating by a rotavap. Finally, the compound was purified using column chromatography with Hexane – Ethyl acetate system (201 mg, 49 % yield).

Synthesis of Compound (4)

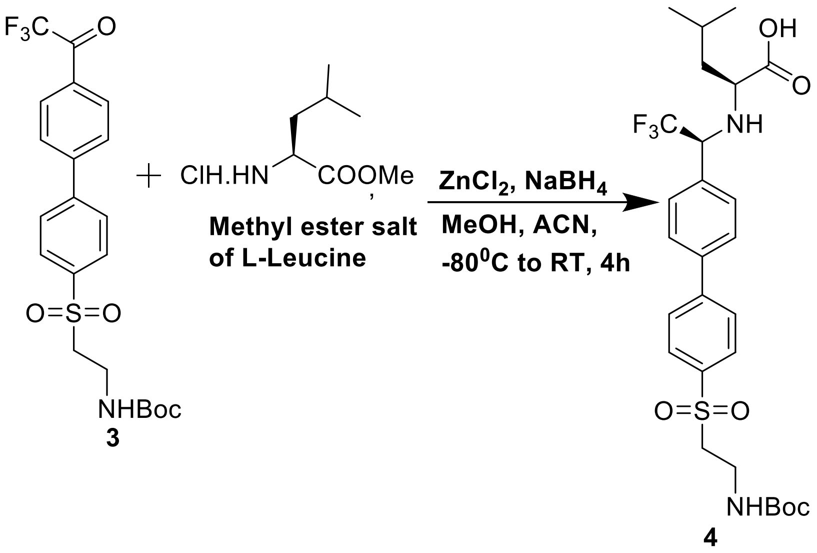

Compound **4** was synthesized according to the reported procedure in the literature with some modifications. Sodium borohydride (0.8872 mmol, 4 equivalent) was put in a round-bottom with anhydrous zinc chloride (2 equivalent) in dry DME at 0º C and allowed to stir at RT for 18 h in a nitrogen atmosphere. Meanwhile, compound **3** (100 mg, 0.218 mmol, 1 equivalent) was put in a round-bottom flask with methyl ester salt of L-Leucine (1.25 equivalent), and potassium carbonate (3 equivalent) in dry methanol, the mixture was warmed to 55º C and kept for 12 h in a nitrogen atmosphere. After that, the reaction mixture was allowed to cool to RT and the methanol was evaporated using rotavap. Dry acetonitrile was added to the mixture and transferred to a round-bottom flask containing the mixture of sodium borohydride and zinc chloride very slowly maintaining the temperature at -44º C. The resulting reaction mixture was allowed to stir at this temperature for 4 h. Acetone was added to the reaction to quench the reducing reagent present in the reaction mixture. Then the reaction mixture was allowed to reach RT for 1 h. HCl (1 N) was added and the solvent was evaporated using an evaporator.

Tertiary butyl methyl ether was added to the crude and extracted using water. The ether layer was collected and dried in an evaporator. The crude was then subjected to preparative reverse phase HPLC purification using a water acetonitrile gradient system. The purified compound was finally freeze-dried before it was used for experimental purposes (50 mg, 40 % yield). Calculated mass [M] = 572.22, found in LCMS = 572.83.

Synthesis of Compound (5)

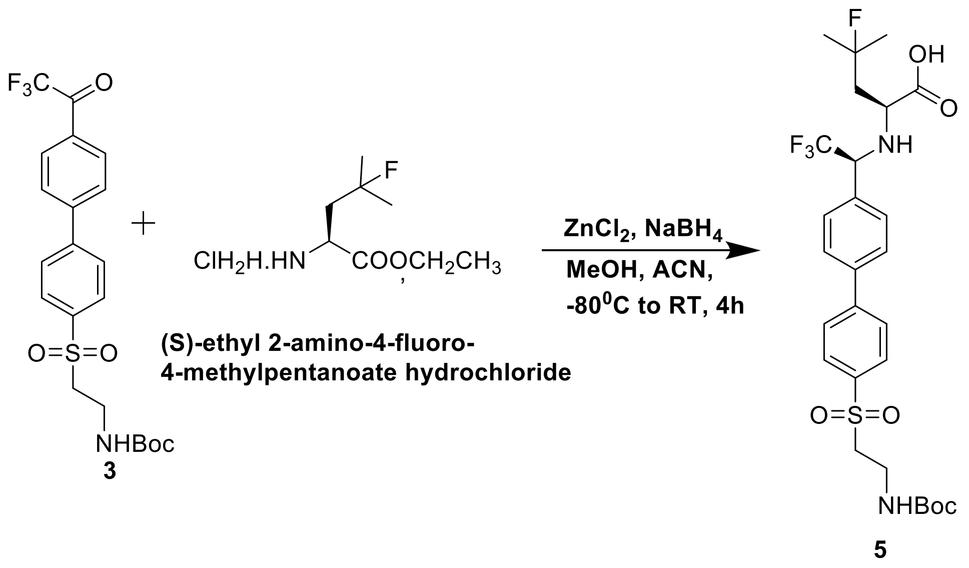

Compound **5** was synthesized according to the reported procedure in the literature with some modifications. Sodium borohydride (0.872 mmol, 4 equivalent) was put in a round-bottom flask with anhydrous zinc chloride (2 equivalent) in dry DME at 0° C and allowed to stir at RT for 18 h in a nitrogen atmosphere. Meanwhile, compound **3** (100 mg, 0.218 mmol, 1 equivalent) was put in a round-bottom flask with (S)-ethyl 2-amino-4-fluoro-4-methyl pentanoate hydrochloride (1.25 equivalent), and potassium carbonate (3 equivalent) in dry methanol and warmed at 55° C for 12 h in a nitrogen atmosphere. After that, the reaction mixture was allowed to cool to RT and the methanol was evaporated using a rotavap. Dry acetonitrile was added to the mixture and transferred to the round-bottom flask containing the mixture of sodium borohydride and zinc chloride very slowly maintaining the temperature at -44° C. The resulting

reaction mixture was allowed to stir at this temperature for 4 h. Acetone was added to the reaction to quench the reducing reagent present in the reaction mixture. Then the reaction mixture was allowed to reach RT for 1 h. HCl (1 N) was added, and the solvent was evaporated using a rotavap. Tertiary butyl methyl ether was added to the crude and extracted. The ether layer was collected and dried in a rotavap. The crude was then subjected to preparative reverse phase HPLC purification using a water acetonitrile gradient system. The purified compound was finally freeze-dried before it was used for experimental purposes (30 mg, 23 % yield). Calculated mass [M] = 590.21, found in LCMS = 590.61.

Synthesis of Compound (7)

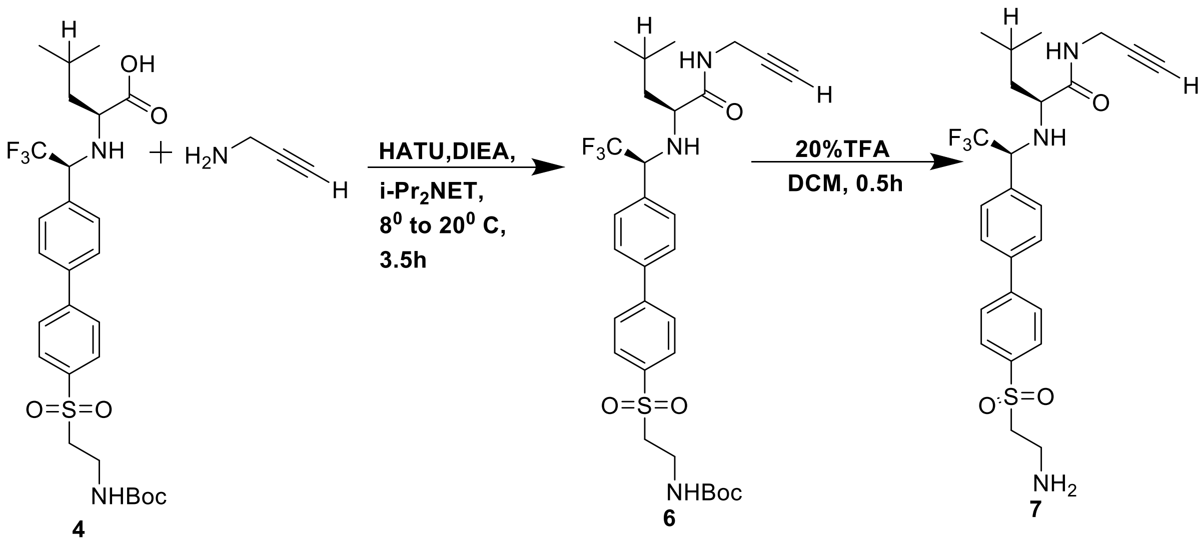

Compound **4,** 50 mg, 0.087 mmol, 1 equivalent, was taken in an ice-cooled dimethylacetamide (DMAc) solution in a round bottomed flask. Propargyl amine (3 equivalent) and HATU (4 equivalent) were discharged in the solution. After 10 mins of stirring at 0° C, DIEA (6 equivalent) was added to the solution. The reaction mixture was allowed to stir for another 2.5 h, keeping the temperature around 8° C. The required amount of water was added to the reaction mixture and allowed to stir for another 1.5 h at 20° C. The precipitate formation was observed at the bottom of the round-bottom flask. The light-yellow colored precipitate was filtered and

washed with a 1:1.2 DMF/water solution, and water. Finally, the washed precipitate was then subjected to preparative reverse phase HPLC purification using a water acetonitrile gradient system. The purified compound **6 (GD30)** was finally freeze-dried before it was used for experimental purposes (24 mg, 45 % yield). Calculated mass [M] = 609.25, found in LCMS = 609.58.

Compound **7,** After purification, compound **6** (21 mg, 0.041 mmol) was Boc deprotected by a 25% TFA solution in DCM giving compound **7**. Compound **7** was purified by preparative reverse phase HPLC using a water acetonitrile gradient system (15 mg, 86 % yield). Calculated mass [M+H] = 510.20, found in LCMS = 510.25.

Synthesis of Compound (8)

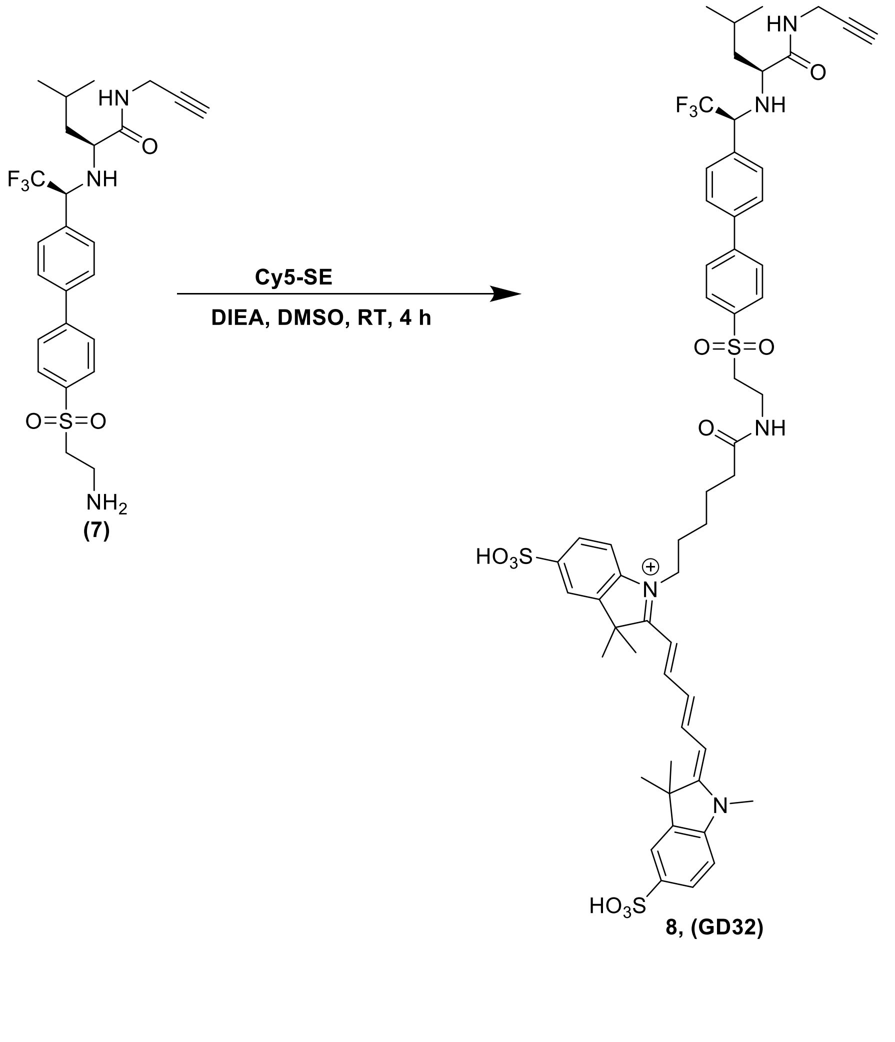

Compound **7** (8 mg, 0.157 mmol, 1 equivalent), Cy5 succinimide ester (1.2 equivalent), and DIEA (5 equivalent) were taken in dry DMSO and allowed to stir at RT for 4 h in an inert dark atmosphere. Compound **8** (**GD32**) was obtained as a dark blue solid after preparative reverse phase HPLC purification of crude using a water acetonitrile gradient system (8 mg, 45 % yield). Calculated mass [M] = 1134.43, found in LCMS = 1134.83.

Synthesis of Compound (9)

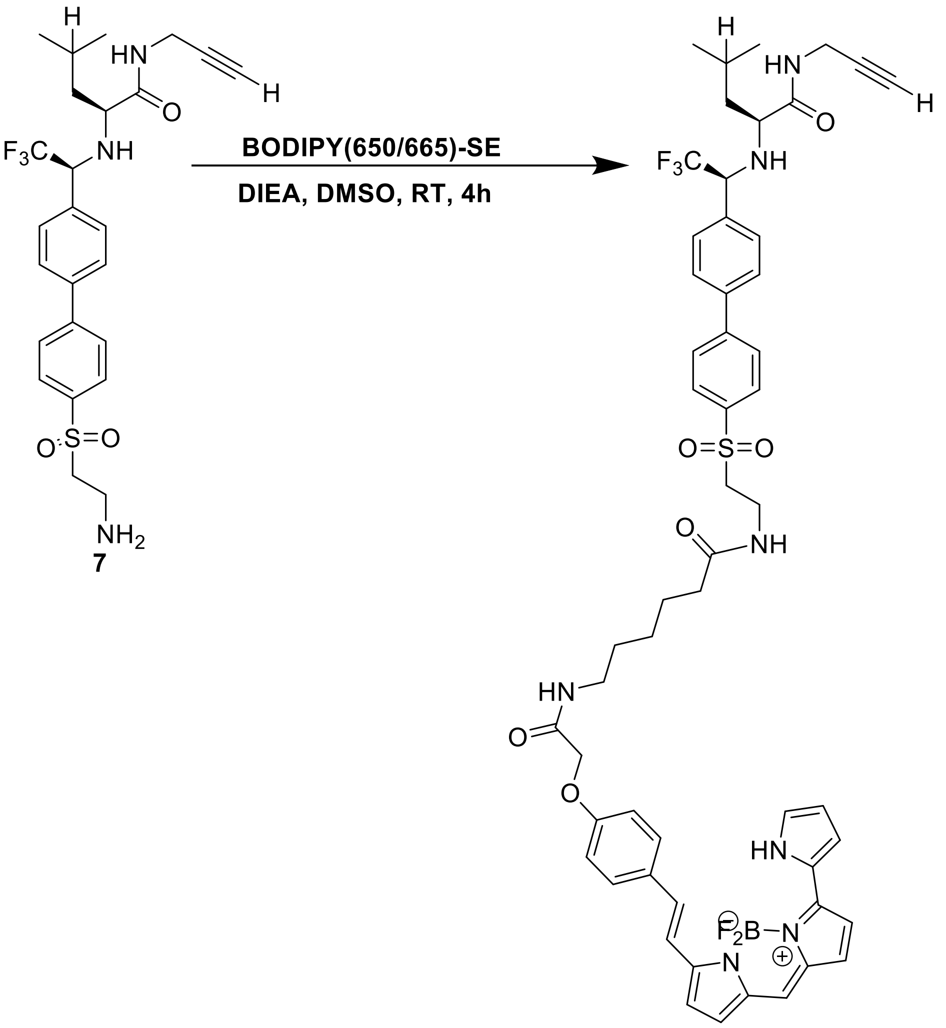

Compound **7** (7 mg, 0.0137 mmol, 1 equivalent), BODIPY (650/665) succinimide ester (1.2 equivalent), and DIEA (5 equivalent) were taken in dry DMSO and allowed to stir at RT for 4 h in an inert dark atmosphere. Compound **9** (**GD36**) was obtained as a dark purple solid after

preparative reverse phase HPLC purification of crude using a water acetonitrile gradient system (3 mg, 21 % yield). Calculated mass [M+H] = 1038.42, found in LCMS = 1038.17.

Synthesis of Compound (12)

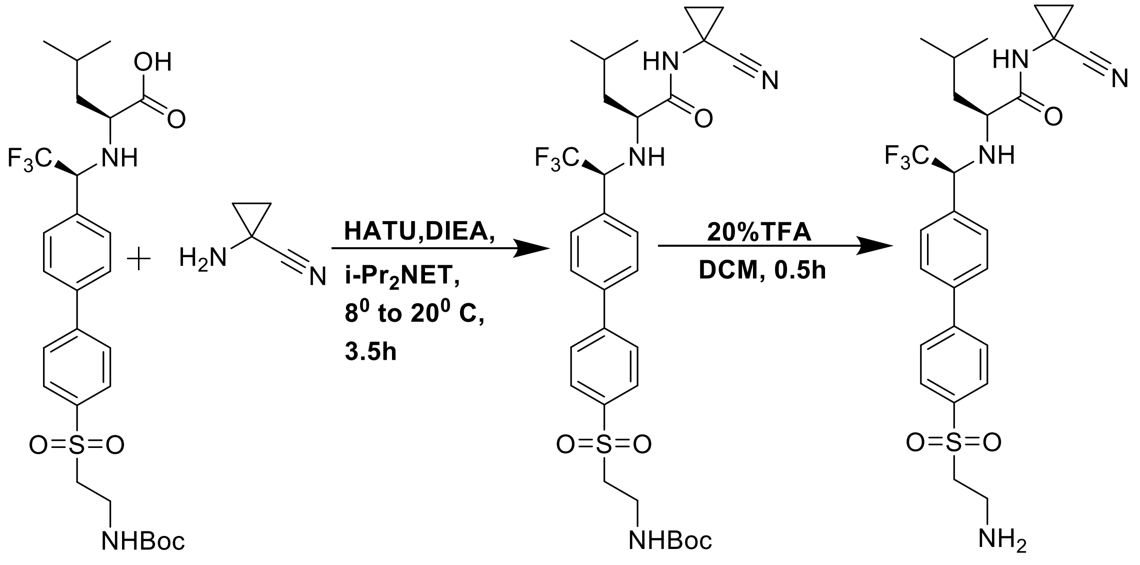

4 **10** **12**

Compound **12** was synthesized using the same procedure which was used to obtain compound **7**. Instead of propargyl amine, 1-aminocyclopropane carbonitrile (3 equivalent) was used with compound **4** (50 mg, 0.08 mmol, 1 equivalent). The desired compound was purified by preparative reverse phase HPLC purification of crude using a water acetonitrile gradient system. (22 mg, 40 % yield). Calculated mass [M-Boc+H] = 537.26, found in LCMS = 537.25.

After purification, compound **10 (**21 mg**,** 0.039 mmol) was Boc deprotected by a 25% TFA solution in DCM to achieve compound **12**. Compound **12** was purified by preparative reverse phase HPLC using a water acetonitrile gradient system (15.7 mg, 89 % yield). Calculated mass [M+H] = 537.21, found in LCMS = 537.25.

Synthesis of Compound (13)

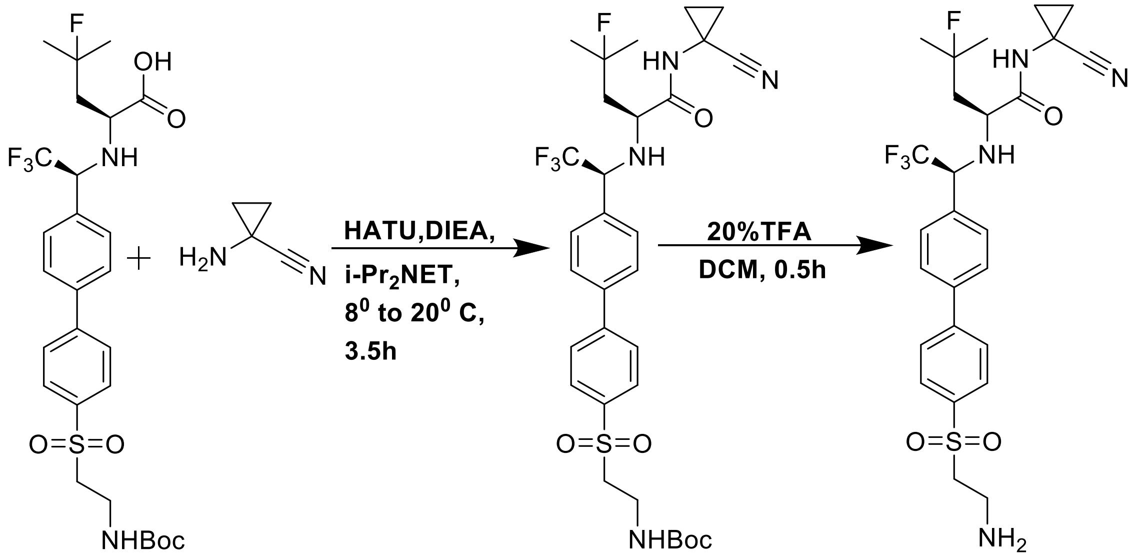

Compound **5** (30.4 mg, 0.051 mmol, 1 equivalent) was taken in an ice-cooled solution of dimethylacetamide (DMAc) in a round-bottom flask. 1-aminocyclopropane carbonitrile (3 equivalent) and HATU (4 equivalent) were discharged in the solution. After 10 mins of stirring at 0° C, DIEA (6 equivalent) was added to the solution. The reaction mixture was allowed to stir for another 2.5 h keeping the temperature around 8° C. The required amount of water was added to the reaction mixture and allowed to stir for another 1.5 h at 20° C. The precipitate formation was observed at the bottom of the flask. The light-yellow colored precipitate was filtered and washed with a 1:1.2 DMF/water solution, and water. Finally, the washed precipitate was then subjected to preparative reverse phase HPLC purification using a water acetonitrile gradient system. The purified compound was finally freeze-dried before it was used for experimental purposes (9 mg, 26.71 % yield). Calculated mass [M+Na] = 677.25, found in LCMS = 676.83.

After purification, compound **11** (7.5 mg, 0.011 mmol) was Boc deprotected by a 25 % TFA solution in DCM to achieve compound **13**. Compound **13** was purified through preparative

reverse phase HPLC purification using a water acetonitrile gradient system (6 mg, 90 % yield). Calculated mass [M] = 554.2, found in LCMS = 554.92.

Synthesis of compound 14

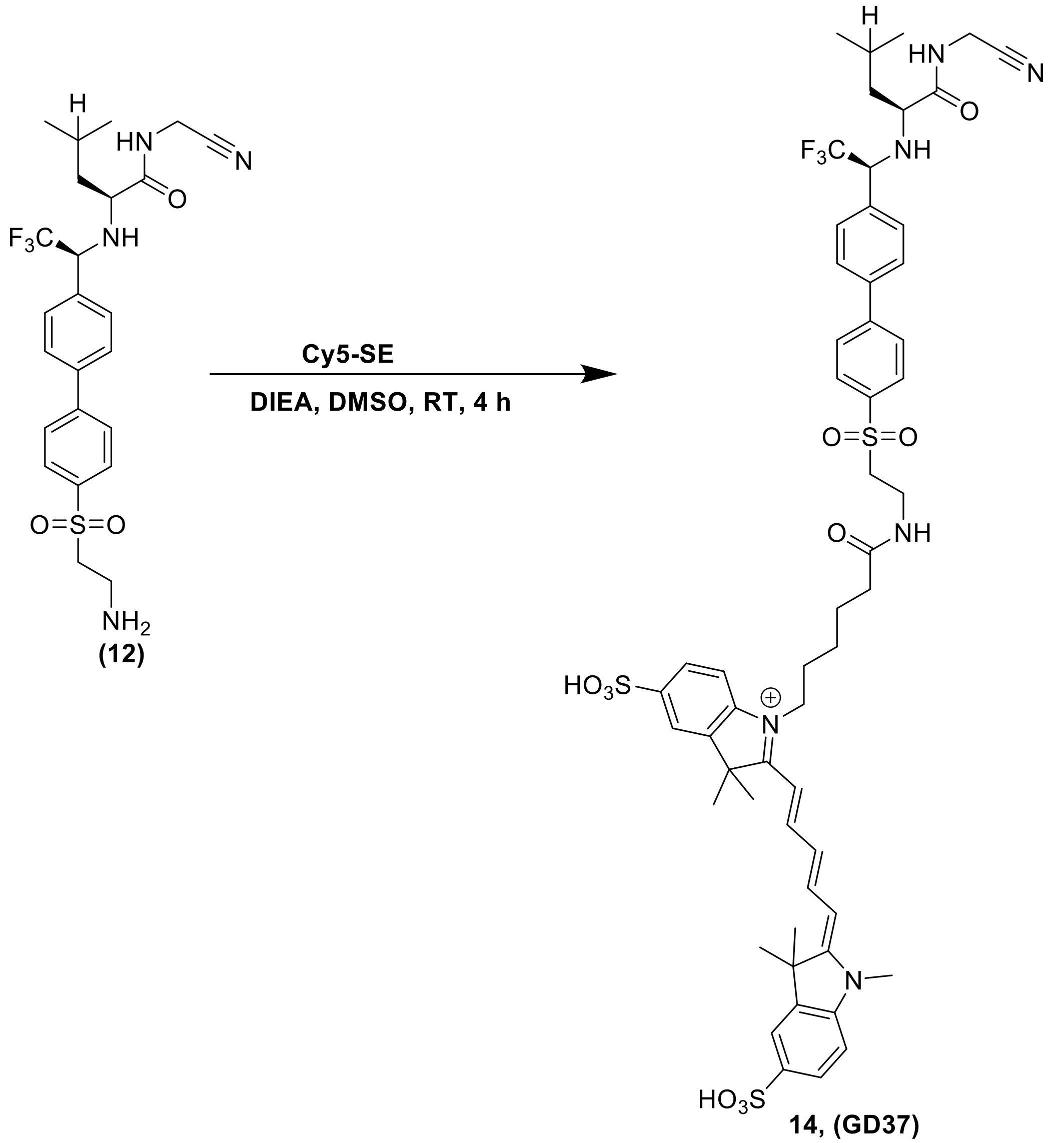

(12)

14, (GD37)

Compound **12** (10 mg, 0.018 mmol, 1 equivalent), Cy5 succinimide ester (1.2 equivalent), and DIEA (5 equivalent) were taken in dry DMSO and allowed to stir at RT for 4 h in an inert dark atmosphere. Compound **14 (GD37)** was obtained as a dark blue solid after preparative reverse phase HPLC purification of crude using a water acetonitrile gradient system (5 mg, 23 % yield). Calculated mass [M] = 1161.41, found in LCMS = 1161.58.

Synthesis of compound 15

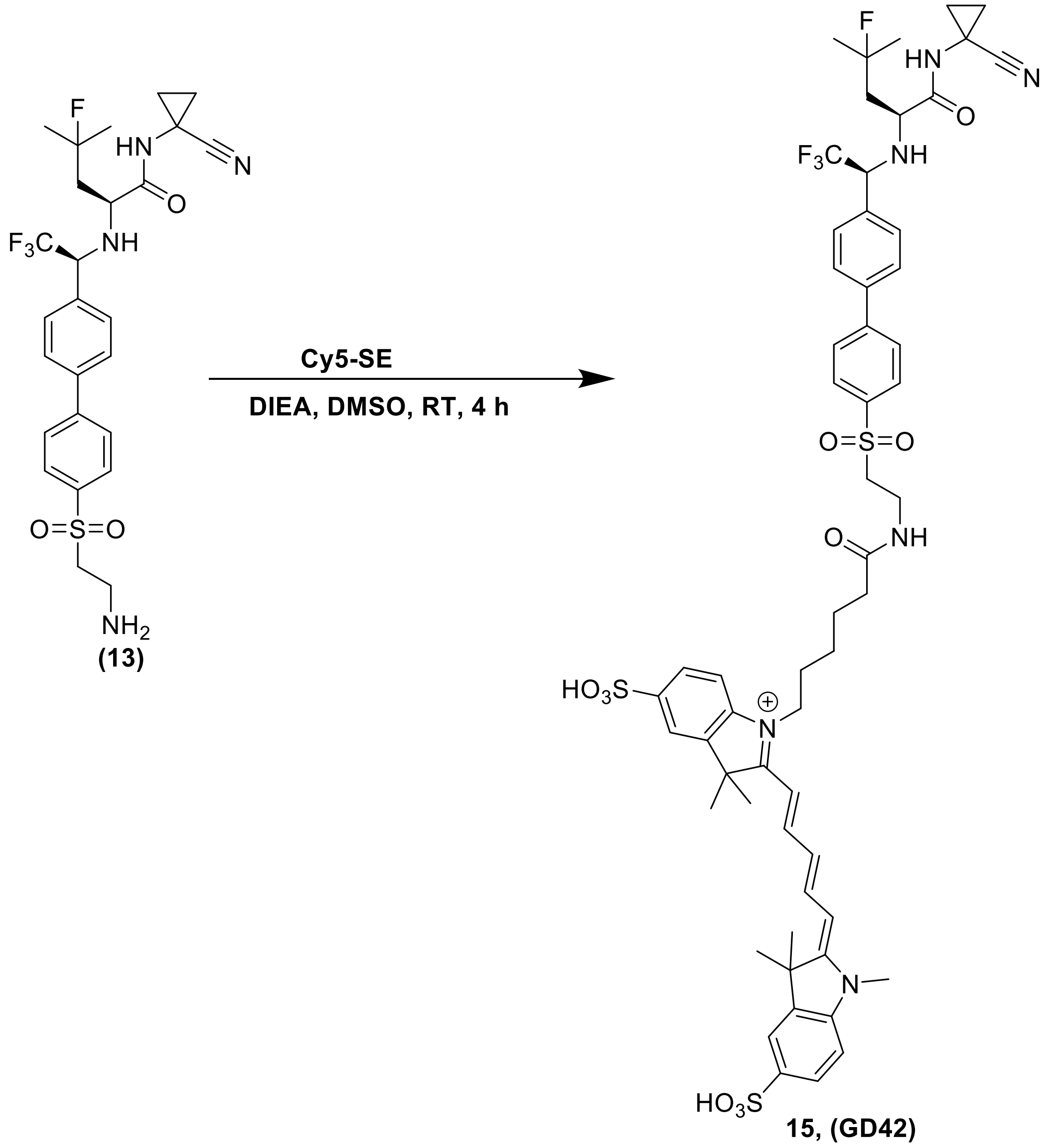

Compound **13** (6 mg, 0.01 mmol, 1 equivalent), Cy5 succinimide ester (1.2 equivalent), and DIEA (5 equivalent) were taken in dry DMSO and allowed to stir at RT for 4 h in the inert dark atmosphere. Compound **15 (GD42)** was obtained as a dark blue solid after preparative reverse phase HPLC purification of crude using a water acetonitrile gradient system (1.2 mg, 9 % yield). Calculated mass [M] = 1177.39, found in LCMS = 1176.92.

Spectroscopic details of compound 2:

^{1}H NMR (500 MHz, $CDCl_3$): δ 7.76 (d, J=5 Hz, 2H), 7.70 (d, J=5 Hz, 2H), 5.21 (s, H), 3.51 (t, J=5 Hz, 2H), 3.31 (t, J=7.5 Hz, 2H), 1.38 (s, 9H).

^{13}C NMR (125 MHz, $CDCl_3$): δ 180.32, 155.84, 145.95, 144.99, 139.31, 131.12, 128.98, 128.64, 128.1, 127.25, 113.33 (q, J=290 Hz), 80.27, 56.1, 34.92, 28.49.

MALDI (ESI): Calc. for $C_{13}H_{18}BrNO_4S$ [M + Na] = 386, found in MALDI = 386.63.

Physical Properties: White-colored solid.

Spectroscopic details of compound 3:

^{1}H NMR (500 MHz, $CDCl_3$): δ 8.17 (d, J=5 Hz, 2H), 8 (d, J=10 Hz, 2H), 7.8 (d, J=10 Hz, 2H), 7.76 (d, J=10 Hz, 2H), 3.54 (t, J=7.5 Hz, 2H), 3.35 (t, J=5 Hz, 2H), 1.38 (s, 9H).

^{13}C NMR (125 MHz, $CDCl_3$): δ 180.32, 155.84, 145.95, 144.99, 139.31, 131.12, 128.98, 128.64, 128.1, 127.25, 113.33 (q, J=290 Hz), 80.27, 56.1, 34.92, 28.49.

MALDI (ESI): Calc. for $C_{21}H_{22}F_3NO_5S$ [M + Na] = 480.11, found in MALDI = 480.23.

Physical Properties: Whitish yellow solid.

Compound 4

^{1}H NMR (500 MHz, $CDCl_3$): δ 7.95 (d, J=5 Hz, 2H), 7.73 (d, J=10 Hz, 2H), 7.58 (d, J=10 Hz, 2H), 7.52 (d, J=5 Hz, 2H), 5.9 (b, 2H), 5.31 (s, 1H), 4.16 (q, J=5 Hz, 1H), 3.57 (q, J=5 Hz, 2H), 3.48 (s, 1H), 3.3 (t, J=5 Hz, 2H), 1.97-1.89 (m, 1H), 1.58-1.47 (m, 2H), 1.41 (s, 9H), 0.93 (dd, J=5 Hz, 6H).

^{13}C NMR (125 MHz, $CDCl_3$): δ179.76, 155.89, 146.20, 139.96, 138, 135.41, 129.28, 128.65, 128.26, 127.87, 124.43 (q, J=280 Hz), 80.38, 62.75 (q, J=28.75 Hz), 58.51, 56.09, 42.72, 34.81, 28.42, 24.88, 23.02, 21.84.

Physical Properties: White-colored solid.

Compound 5:

^{1}H NMR (500 MHz, $CDCl_3$): δ 7.95 (d, J=8 Hz, 2H), 7.73 (d, J=8 Hz, 2H), 7.59 (d, J=8 Hz, 2H), 7.5 (d, J=8 Hz, 2H), 5.29 (s, 1H), 5.26 (b, 1H), 4.28 (q, J=7 Hz, 1H), 3.68 (dd, J=4 Hz, 1H), 3.57 (d, J=5 Hz, 2H), 3.3 (t, J=6 Hz, 2H), 2.22-2.136 (m, 2H), 2-1.92 (m, 1H), 1.47 (d, J=10.5 Hz, 3H), 1.43 (d, J=10.5 Hz, 3H), 1.408 (s, 9H),

^{13}C NMR (125 MHz, $CDCl_3$): δ176.87, 146.12, 140.2, 138.09, 134.49, 129.39, 128.73, 128.25, 128.05, 124.28 (q, J=280 Hz), 94.9 (d, J=165 Hz), 80.24, 62.42 (q, J=30 Hz), 56.66, 56.13, 43.97, 34.83, 28.45, 26.96 (q, J=23.75 Hz).

Physical Properties: White-colored solid.

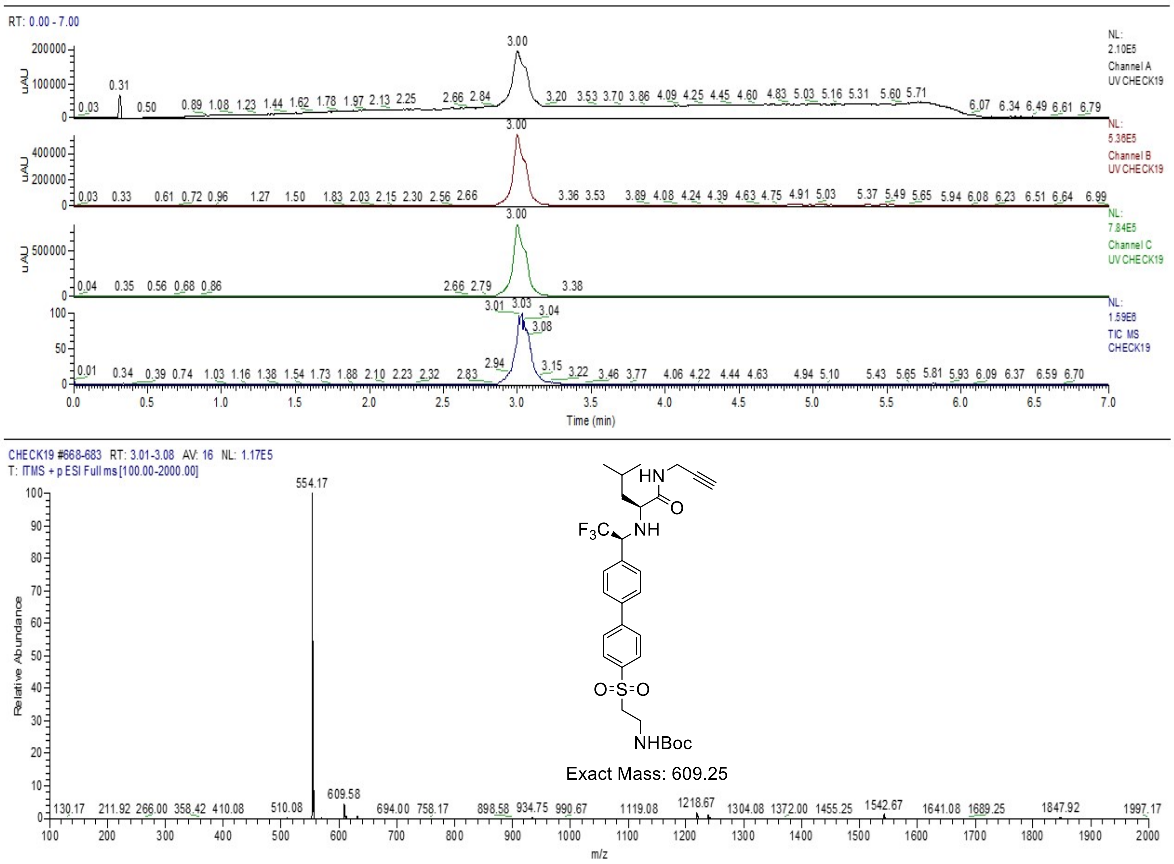

Figure S10. LCMS spectra of compound **6**.

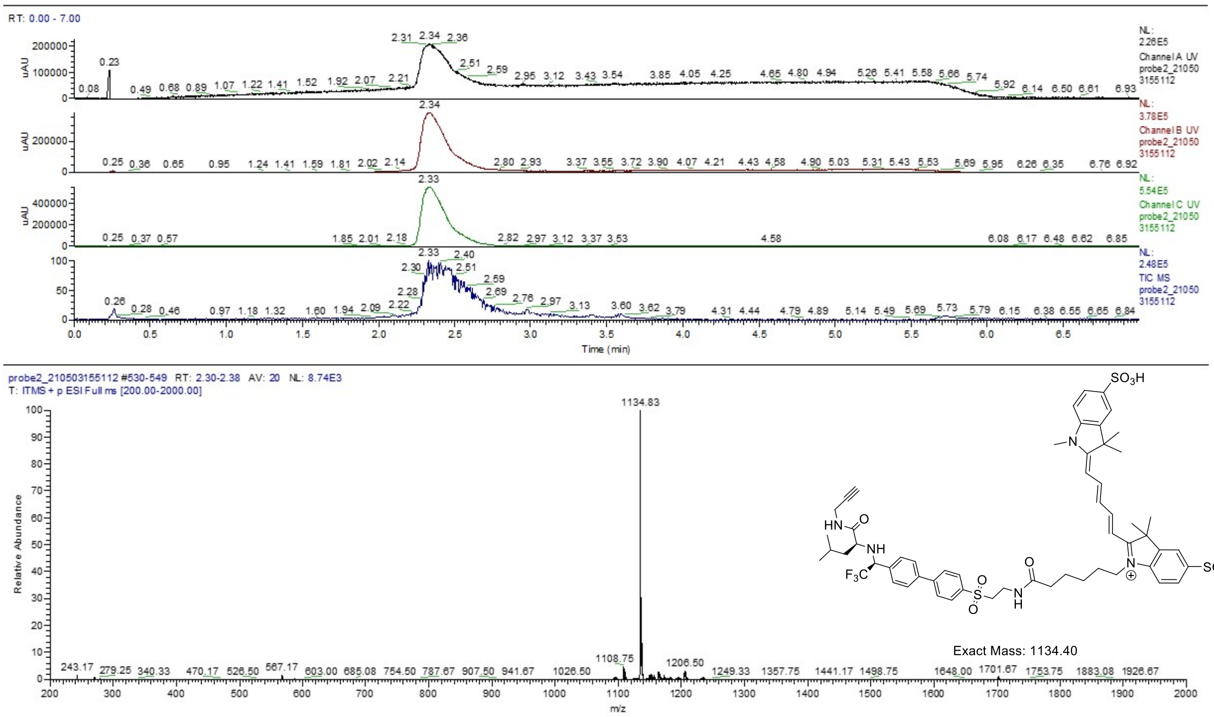

Figure S11. LCMS spectra of compound **8 (GD32)**.

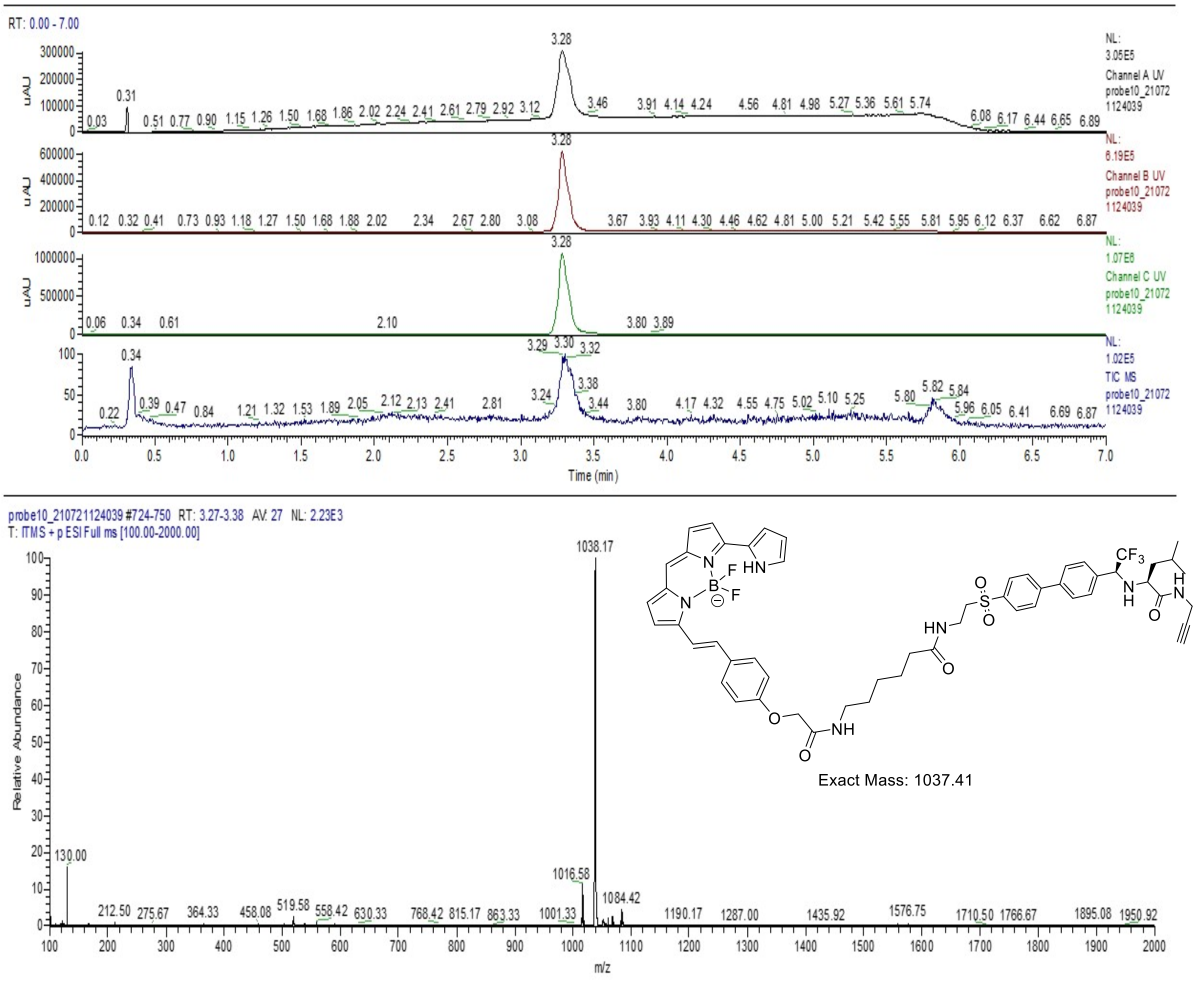

Figure S12. LCMS spectra of compound **9 (GD36)**.

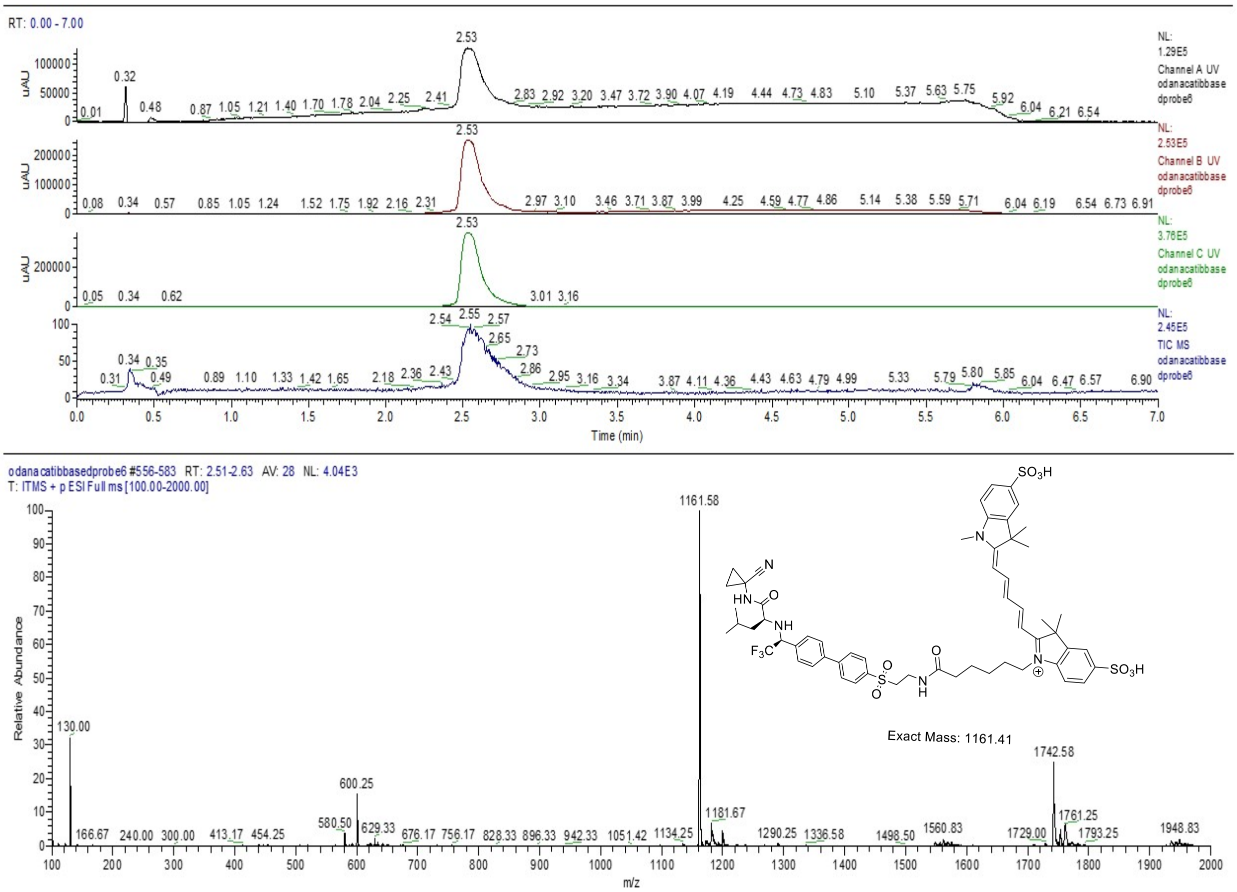

Figure S13. LCMS spectra of compound **14 (GD37)**.

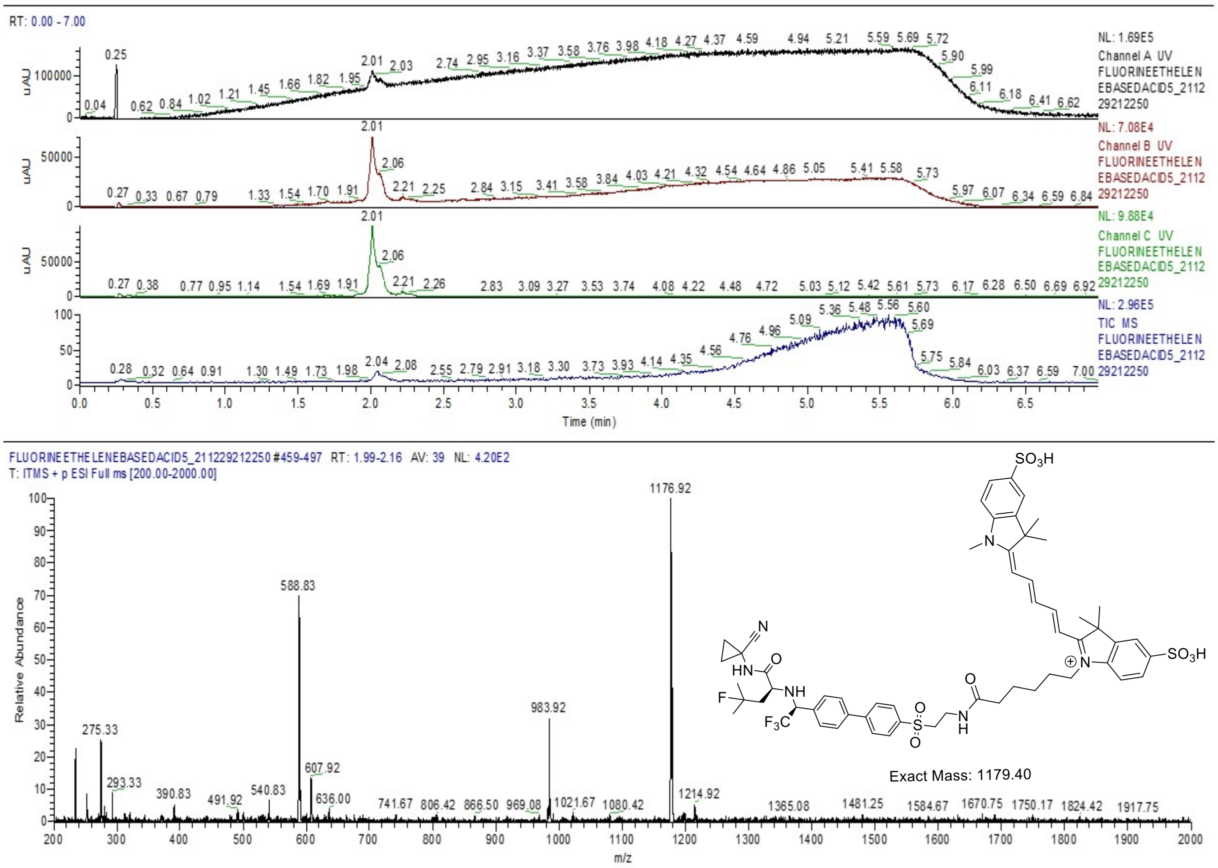

Figure S14. LCMS spectra of compound **15 (GD42)**.

References

[1] D. Bromme, F. S. Nallaseth, B. Turk, *Methods* **2004**, 32, 199–206.

[2] R. Smoum, A. Bar, B. Tan, G. Milman, M. Attar-Namdar, O. Ofek, J. M. Stuart, A. Bajayo, J. Tam, V. Kram, D. O'Dell, M. J. Walker, H. B. Bradshaw, I. Bab, R. Mechoulam, *Proc. Natl. Acad. Sci.* **2010**, 107, 17710–17715.

[3] G. Blum, S. R. Mullins, K. Keren, M. Fonovič, C. Jedeszko, M. J. Rice, B. F. Sloane, M. Bogyo, *Nat. Chem. Biol.* **2005**, 1, 203–209.

[4] H. Ben-Bassat, D. V. Vardi, A. Gazit, S. N. Klaus, M. Chaouat, Z. Hartzstark, A. Levitzki, *Exp. Dermatol.* **1995**, *4*, 82–88.
